# Identification and functional analyses of sex determination genes in the sexually dimorphic stag beetle *Cyclommatus metallifer*

**DOI:** 10.1186/s12864-016-2522-8

**Published:** 2016-03-22

**Authors:** Hiroki Gotoh, Robert A. Zinna, Ian Warren, Michael DeNieu, Teruyuki Niimi, Ian Dworkin, Douglas J. Emlen, Toru Miura, Laura C. Lavine

**Affiliations:** Department of Entomology, Washington State University, Pullman, WA 99164 USA; Graduate School of Environmental Science, Hokkaido University, Sapporo, Hokkaido 060-0810 Japan; Department of Zoology, Michigan State University, East Lansing, MI 48824 USA; Graduate School of Bioagricultural Sciences, Nagoya University, Nagoya, Aichi 464-8601 Japan; Division of Evolutionary Developmental Biology, National Institute for Basic Biology, Okazaki, Aichi 444-8585 Japan; Department of Biology, McMaster University, Hamilton, ONT L8S 4K1 Canada; Division of Biological Sciences, University of Montana-Missoula, Missoula, MT 59812 USA

**Keywords:** *Cyclommatus metallifer*, Sex determination, Sexual dimorphism, Sexual selection

## Abstract

**Background:**

Genes in the sex determination pathway are important regulators of sexually dimorphic animal traits, including the elaborate and exaggerated male ornaments and weapons of sexual selection. In this study, we identified and functionally analyzed members of the sex determination gene family in the golden metallic stag beetle *Cyclommatus metallifer,* which exhibits extreme differences in mandible size between males and females.

**Results:**

We constructed a *C. metallifer* transcriptomic database from larval and prepupal developmental stages and tissues of both males and females. Using Roche 454 pyrosequencing, we generated a *de novo* assembled database from a total of 1,223,516 raw reads, which resulted in 14,565 isotigs (putative transcript isoforms) contained in 10,794 isogroups (putative identified genes). We queried this database for *C. metallifer* conserved sex determination genes and identified 14 candidate sex determination pathway genes. We then characterized the roles of several of these genes in development of extreme sexual dimorphic traits in this species. We performed molecular expression analyses with RT-PCR and functional analyses using RNAi on three *C. metallifer* candidate genes – *Sex-lethal* (*CmSxl*)*, transformer-2* (*Cmtra2*), and *intersex* (*Cmix*). No differences in expression pattern were found between the sexes for any of these three genes. In the RNAi gene-knockdown experiments, we found that only the *Cmix* had any effect on sexually dimorphic morphology, and these mimicked the effects of *Cmdsx* knockdown in females. Knockdown of *CmSxl* had no measurable effects on stag beetle phenotype, while knockdown of *Cmtra2* resulted in complete lethality at the prepupal period. These results indicate that the roles of *CmSxl* and *Cmtra2* in the sex determination cascade are likely to have diverged in stag beetles when compared to *Drosophila.* Our results also suggest that *Cmix* has a conserved role in this pathway. In addition to those three genes, we also performed a more complete functional analysis of the *C. metallifer dsx* gene (*Cmdsx*) to identify the isoforms that regulate dimorphism more fully using exon-specific RNAi. We identified a total of 16 alternative splice variants of the *Cmdsx* gene that code for up to 14 separate exons. Despite the variation in RNA splice products of the *Cmdsx* gene, only four protein isoforms are predicted. The results of our exon-specific RNAi indicated that the essential CmDsx isoform for postembryonic male differentiation is CmDsxB, whereas postembryonic female specific differentiation is mainly regulated by CmDsxD.

**Conclusions:**

Taken together, our results highlight the importance of studying the function of highly conserved sex determination pathways in numerous insect species, especially those with dramatic and exaggerated sexual dimorphism, because conservation in protein structure does not always translate into conservation in downstream function.

**Electronic supplementary material:**

The online version of this article (doi:10.1186/s12864-016-2522-8) contains supplementary material, which is available to authorized users.

## Background

From the colorful tail feathers of peacocks to the enormous antlers of deer, exaggerated sexual structures are ubiquitous across the animal kingdom [1–6]. Among the most extreme examples of sexually-selected traits are the weapons of beetles. Many species of scarab beetles have horns jutting out from the head and thorax and many stag beetles have elongated mandibles [6–8]. All are used as weapons for male-male combat over access to reproduction. The shapes and sizes of beetle weapons differ widely among families, genera, and sometimes even between populations of the same species, and most of these weapons are sexually dimorphic [9, 10].

Although much research has focused on the function and evolution of beetle weapons (e.g. [11–14]), the proximate mechanisms regulating their development are also beginning to be explored. Recent advances in next-generation sequencing technologies and the successful utilization of RNA interference (RNAi) have enabled researchers to delve into the genetic mechanisms underlying the growth, expression, and condition-dependence of these traits. Several recent studies focusing on horns in scarab beetles have revealed many of the genetic mechanisms regulating horn development, growth, and condition-dependence [15–17]. Because of their sex-specific expression, the sex determination genes of insects, which are highly conserved across the arthropods and animals in general [18], are particularly important to understanding the development of sexually selected weapon traits [17].

Despite the overall conservation in sexual differentiation genes in arthropods, the initial cues of the sex determination cascade are highly diverse both at the chromosomal level (e.g. XX-XY in *Drosophila*, ZW-ZZ in Lepidoptera, XX-XO in aphids, and haplodiploidy in Hymenoptera) and at the level of the molecular signal (e.g. sex-dependent activation of the *Sex-lethal* (*Sxl*) gene in *Drosophila* [18, 19], *transformer* (*tra*) in *Ceratitis* [20, 21], and sex-specific expression of piRNA *fem* in *Bombyx* [22]). Diversity among species is also found in the maternal transfer of *tra* mRNA in *Nasonia* [23] and heterozygosity in the *complementary sex determiner* (*csd*) gene’s activation of the gene *feminizer* (*fem*) in honeybees [24]. Although the relative importance of these upstream signals appears to vary from species to species, as does the default activation state of the *tra* and *fem* genes (ON in females versus OFF in males), all sex determination mechanisms known for insects thus far integrate expression of this same network of genes [18], and they all result in the sex-specific expression of alternative splice variants of the sex determination gene *doublesex* (*dsx*), which, in turn, appears to trigger sex-specific patterns of growth of dimorphic body parts [18, 25–27].

Recently several authors, including our group, have shown that *dsx* regulates sex-specific trait expression in beetles, including species with horns and elongated mandibles [28–31]. However, very little is known about the initial cues of sex determination in these species, or about pathway members acting upstream of *dsx* in the sex determination cascade.

**Fig. 1:**
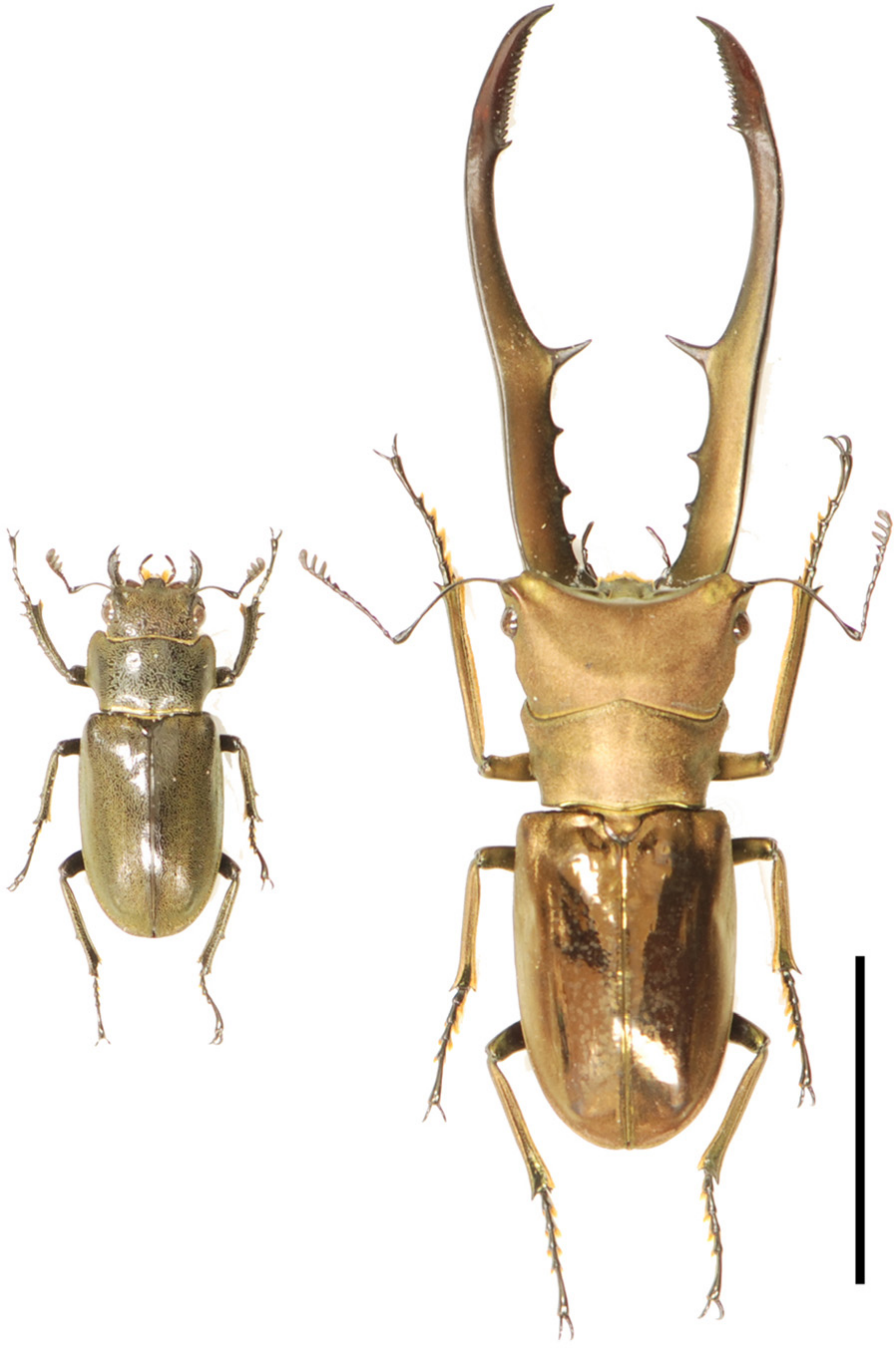
The golden metallic stag beetle, *Cyclommatus metallifer*. Female (left) and large male (right) are shown. Only males possess enlarged mandibles, which are used for male-male competition over resources. Scale bar indicates 20 mm

In this study, we tested the role of several genes in the sex determination pathway known to regulate sex-specific trait expression in other animals in the sexually dimorphic and male dimorphic golden metallic stag beetle *Cyclommatus metallifer* (Fig. 1). This species (and many other species of Lucanidae) possess extremely elongated mandibles, which are expressed in a sex and condition dependent manner [31]. We constructed a transcriptome for the critical prepupal period of development when all adult structures are determined and differentiating in *C. metallifer* in order to rigorously test the hypothesis that the effector genes for sexual differentiation are conserved with other insects*.* We queried this transcriptome database for 24 sex-determination cascade genes known in *Drosophila,* and identified 14 putative homologs in *C. metallifer*. From these 14 sex-determination cascade gene homologs, we selected three central sex-determination cascade genes for further analyses; *tra, tra2,* and *ix,* which have significant roles during sexual differentiation in many insects. Also, we analyzed *Sxl,* which is recruited as a key upstream switch for sex determination in the *Drosophila* lineage.

The gene *Sxl* is activated in *Drosophila* females based on the dose of X chromosome [18, 32]. Once activated, female splice forms of *Sxl* appear to control their own expression by a positive feedback splicing mechanism, resulting in the regulation and splicing of downstream genes such as *tra* and *dsx* in a sex-specific manner [33]. The *Sxl* gene is not activated in *Drosophila* males, and, in the absence of this signal, male-specific *tra* and *dsx* splice variants are expressed. The role of *Sxl* in sexual differentiation appears to be specific to the *Drosophila* lineage, though the functions of *Sxl* outside of *Drosophila* are not well known [18, 34].

The *ix* gene is also necessary for female development in *Drosophila* and has been shown to act in concert with *dsx* in the sex determination pathway [35, 36]. *tra* and *tra2* are required for female sexual development in many insect groups including *Drosophila, Apis* and *Tribolium,* and function by regulating the alternative splicing of *dsx* [21, 33, 37, 38]. *tra* is expressed as different splice variants in a sex-specific manner [37] and, as with *Sxl* in *Drosophila*, female specific splice forms of *tra* in some insects contain an autoregulatory domain that appears to result in self-activation of expression, with the resulting positive feedback contributing to sex differences in expression and splice forms of downstream genes in the pathway [21, 37]. What roles these genes may have in regulating sexual dimorphisms in animals with sexually-selected weapons has yet to be explored.

We examined the expression pattern of *C. metallifer tra*, *ix*, *Sxl* and *tra2* (hereafter, *Cmtra*, *Cmix*, *CmSxl* and *Cmtra2*, respectively) using RT-PCR. Also, we analyzed the function of *Cmix*, *CmSxl* and *Cmtra2* by RNA interference (RNAi). Functional analyses revealed that of these, *Cmix* is necessary for female differentiation, while *CmSxl* is not necessary for sexual trait differentiation in either males or females. Knockdown of *Cmtra2* resulted in death at the prepupal stage in both sexes, suggesting that *Cmtra2* likely has an additional function during development besides its known role in sex determination. In this study we also report the functional characterization of male and female specific isoforms of the sex determination gene *C. metallifer doublesex* (*Cmdsx*), which was initially identified in a previous study by our group. Functional analyses via RNAi for isoform-specific *Cmdsx* genes revealed that two have critical roles in sex-specific weapon trait differentiation in *C. metallifer*.

## Results

### Sequencing and assembly

**Fig. 2:**
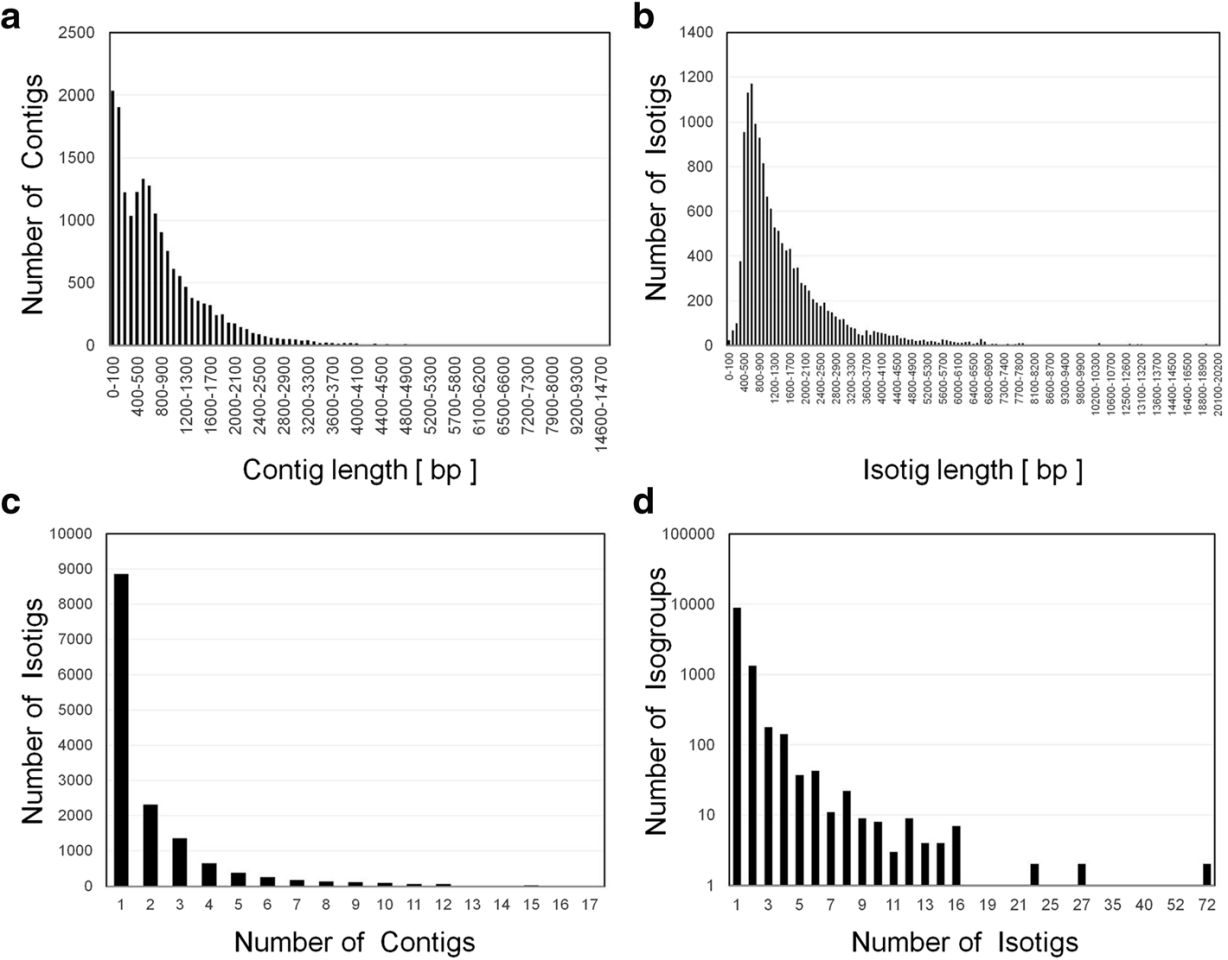
Summary of *Cyclommatus metallifer* transcriptome assembly. **a** Length distribution of contig sequences. X-axis indicates sequence length of contig and Y-axis indicates number of contigs. **b** Length distribution of isotig sequences. X-axis indicates sequence length of isotig and Y-axis indicates number of isotigs (**c**) Number of contigs used in the assembly of individual isotigs. X-axis indicates number of contigs per each isotig and Y-axis indicates number of isotigs (**d**) Number of isotigs used in the assembly of individual isogroups. X-axis indicates number of isotigs per each isogroup and Y-axis indicates number of isogroups

We performed Roche 454-pyrosequencing of an mRNA-enriched RNA library prepared from head and prothorax tissue of *C. metallifer* larvae and prepupae - three each of small and large individuals of both sexes. This stage was chosen as the most likely developmental period when differentiation of adult structures is occurring [31, 39]. A total of 1,223,516 raw reads consisting of 391,313,087 bp were sequenced. Raw sequence data was submitted to NCBI’s Sequence Read Archive (SRA) under the Bioproject Accession number PRJNA311137. Assembled and annotated transcriptome files available upon request. After adapter trimming and quality filtering, the reads were assembled into a *de novo* transcriptome using Newbler 2.9. This transcriptome produced 17,894 contigs, incorporating 353,508,757 base pairs and 1,106,461 reads. 10,468 contigs were larger than 500 bp (labeled as “large contigs” by the Newbler assembler, Fig. 2a, Table 1). The average “large” contig size was 1,267 bp, with a max contig size of 16,848 bp (Fig. 2a). The average read depth was 87.84, and the N50 was 1479 (Table 1). The assembled contigs were used to generate putative isoform variants, or isotigs. 14,565 isotigs were generated, with an average size of 1,592 bp (Fig. 2b). The average number of contigs per isotig was 2.1, with 8,863 isotigs deriving from single contigs (Fig. 2c). The isotigs were grouped into 10,793 unique isogroups (or an estimate of the total number of genes expressed in *C. metallifer* across the tissue libraries). A total of 8,933 isogroups only contained a single isotig, with an average of 1.4 isotigs per isogroup (Fig. 2d).

**Table 1:** Newbler 2.9 assmbly metrics

Stastistic	Newbler metric
Read generated	1223516
Number of aligned reads	1106461 (90.43 %)
Bases generated	387117124
Aligned bases	353508757 (91.32 %)
Contigs generated	17894
“Large” contigs (>500 bp)	10468 (58.5 %)
Isotigs generated	14565
Isogroups generated	10793
Isotigs with BLASTx results (e-5)	12056 (82.77 %)
Isotigs with homology to Tribolium (e-5)	9492 (78.73 %)

**Fig. 3:**
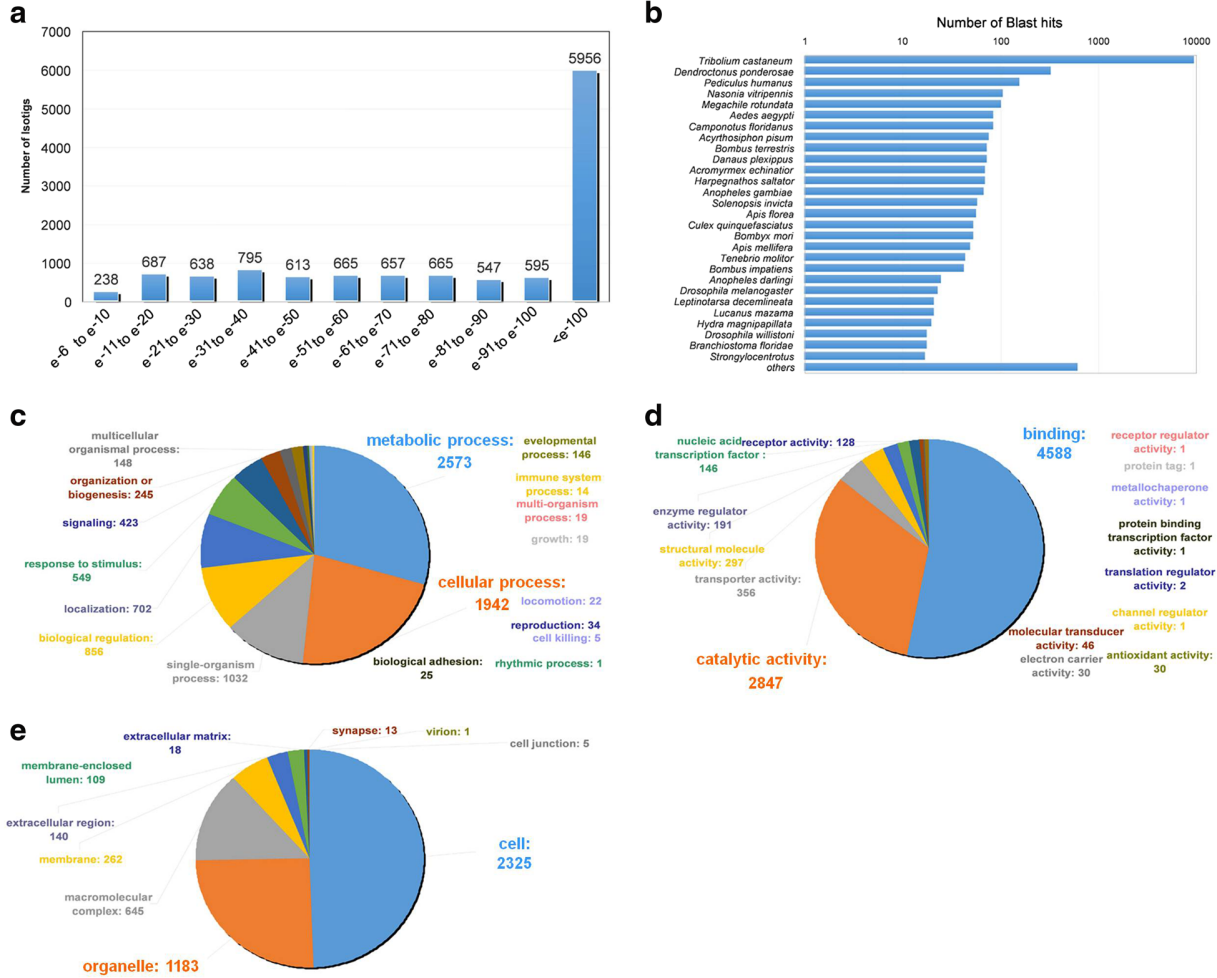
Results of BLASTx search and classification of sequences based on GO term analyses. **a** Distribution of BLASTx top-hits against NCBI non-redundant protein database. E-value cutoff of e-5 was used as threshold. Results with an e-value < e-100 were clustered together. **b** Distribution of BLASTx results, clustered by species of origin. BLASTx hits that returned an “unknown” species were removed from the analysis. **c-e** Gene Ontology isotig groups for Biological Process (**c**), Molecular Function (**d**), and Cellular Component (**e**)

After assembly, we performed BLASTx searches of the isotigs against the NCBI non-redundant protein database [40], using an e-value cut-off of e^−5^. After the BLASTx search, isotigs from each isogroup were filtered by best BLAST hit. If no members of an isogroup had a BLAST hit, one isotig from that isogroup was randomly selected to represent that isogroup. This left a total of 10,772 isotigs. 8,774 isotigs (78.38 %) had significant homology with proteins in the database. Many of them (49.2 %) have e-values < e^−100^ (Fig. 3a). Most of the isotigs (80.77 %) had the highest similarity with sequences of red flour beetle *Tribolium castaneum* (Fig. 3b). Considering their phylogenetic closeness (both *Cyclommatus* and *Tribolium* are in the order Coleoptera) and the completeness of *Tribolium* genome sequence and its annotation, this result is expected. Gene Ontology (GO) terms were assigned to each isotig using Blast2GO (Fig. 3c-e).

### Identification of sex-determination cascade genes

Using homologous gene sequences of *Drosophila* and *Tribolium* as our query terms (Table 2), we searched for 24 candidate genes from the sex-determination cascade (Table 2) against our *C. metallifer* database using BLASTx. A total of 14 *C. metallifer* genes were identified as homologous with known sex determination genes (Table 2) based on e-value (threshold < e^−40^) and/or gene phylogenies (Additional file 1: Figure S1-S14).

**Table 2:** Summary of database search for candidate genes

Candidate gene name (ab)	OrthoDB7_ID	Accession No. of *Drosophila* homolog for query	Accession No. of *Tribolium* homolog for query	Isotig number of the homolog or highest similarity (*Drosophila* query)	Isotig number of the homolog or highest similarity (*Tribolium* query)	e-value (*Drosophila* query)	e-value (*Tribolium* query)	Longest hit length and the percentage of amino acid identity (*Drosophila* query)	Longest hit length and the percentage of amino acid identity (*Tribolium* query)	Identified as *Cyclommatus* homolog of
*groucho (gro)*	EOG7BD0RV	NM_001260380	XM_963022	**7235**	**7235**	0	0	321/355 (90 %)	323/348 (93 %)	***groucho (gro)***
*ovo/shavenbaby (ovo)*	EOG7C0766	NM_001169202	XM_969788	**7894**	**7894**	9.00E-99	5.00E-131	133/157 (85 %)	181/192 (94 %)	***ovo/shavenbaby (ovo)***
*sansfille (snf)*	EOG78WZ6X	NM_078490	XM_963178	**10553**	**10553**	5.00E-88	2.00E-107	85/98 (87 %)	99/106 (93 %)	***sansfille (snf)***
*hopscotch (hop)*	EOG74JNNN	NM_078564	XM_969329	**5920**	**5920**	3.00E-87	0	39/69 (57 %)	194/248 (78 %)	***hopscotch (hop)***
*fruitless (fru)*	EOG7S84VN	NM_079673	NM_001164218	**8868**	**8868**	1.00E-76	3.00E-138	97/125 (78 %)	124/156 (79 %)	***fruitless (fru)***
*virillizer (vir)*	EOG7DRWGS	NM_080161	XM_001807225	**8354**	**7116**	9.00E-54	2.00E-179	62/80 (78 %)	81/126 (64 %)	***virillizer (vir)***
*daughterless (da)*	EOG7BGW18	NM_001273411	XM_968179	**12902**	**12902**	4.00E-53	4.00E-93	89/98 (91 %)	122/126 (97 %)	***daughterless (da)***
*female lethal d (fl(2)d)*	EOG7KT8QP	NM_166010	XM_969964	**4453**	**4453**	1.00E-51	2.00E-116	55/63 (87 %)	116/142 (82 %)	***female lethal d (fl(2)d)***
*deadpan (dpn)*	EOG7X6ZFD	NM_057575	XM_962601	**10698**	**10698**	7.00E-47	1.00E-80	73/118 (62 %)	109/132 (83 %)	***deadpan (dpn)***
*transformer2 (tra2)*	EOG7Z3SMD	NM_057416	XM_963457	**1275**	**1275**	2.00E-41	2.00E-79	59/107 (55 %)	111/120 (93 %)	***transformer2 (tra2)***
*intersex (ix)*	EOG7162KM	NM_136833	XM_008200746	**10843**	**10843**	4.00E-32	4.00E-68	29/47 (62 %)	108/143 (76 %)	***intersex (ix)***
*Sex-lethal (Sxl)*	EOG72ZQVX	NM_001031891	NM_001145943	4016	**10319**	4.00E-48	3.00E-42	63/128 (49 %)	68/100 (68 %)	***Sex-lethal (Sxl)***
*extramacrochaetae (emc)*	EOG7QCMBK	NM_079152	XM_964922	**9148**	**9148**	2.00E-26	1.00E-63	40/70 (57 %)	88/112 (79 %)	***extramacrochaetae (emc)***
*dissatisfaction (dsf)*	EOG7VJ5CF	NM_001273180	XM_970162	6548	6548	3.00E-43	3.00E-51	38/107 (36 %)	41/107 (38 %)	*ultraspiracle (usp)*
*hermaphrodite (her)*	EOG7JXFJ7	NM_001273577	Not found in OrthoDB	9417	NS	4.00E-10	NS	11/34 (32 %)	NS	Hypothetical protein
*degringolade (dgrn)*	EOG7KX50F	NM_141339	Not found in OrthoDB	7560	NS	2.00E-04	NS	12/27 (44 %)	NS	*E3 ubiquitin-protein ligase*
*scute/sisterlessB (sc)*	EOG7HJ870	NM_057455	Not found in OrthoDB	9495	NS	0.01	NS	14/24 (58 %)	NS	*delilah (dei)*
*ovarian tumor (otu)*	EOG7N9B8P	NM_001272403	EEZ98091	1660	1444	0.02	0.65	16/46 (35 %)	11/37 (30 %)	Hypothetical protein/*terribly reduced optic lobes (trol)*
*standstill (stil)*	EOG7RVNX6	NM_057404	Not found in OrthoDB	3203	NS	0.06	NS	11/34 (32 %)	NS	*Brain-enriched guanylate kinase-associated protein*
*transformer (tra)*	EOG7HN4HG	NM_079390	JQ857102	2890	**4317**	0.12	2.00E-08	10/27 (37 %)	17/41 (41 %)	*mitochondrial ribosomal protein L1/* ***transformer (tra)***
*runt (run)*	EOG73RPRJ	NM_078700	XM_964184	7872	5910	0.28	0.26	14/33 (42 %)	11/18 (61 %)	*ribosomal protein L4e/egalitarian (egl)*
*doublesex (dsx)*	EOG77DWNS	NM_169202	XM_001807396	2520	6093	0.55	0	10/19 (53 %)	16/31 (52 %)	*NSFL1 cofactor p47/Ubiquinol-cytochrome c reductase core protein 1*
*sisterless-A (sisA)*	EOG7FZBDN	NM_078561	Not found in OrthoDB	6705	NS	0.66	NS	17/45 (38 %)	NS	*glypican-4*
*outstreched (os)*	EOG7TJFZ9	NM_001103545	Not found in OrthoDB	3953	NS	0.85	NS	11/30 (37 %)	NS	*High mobility group protein DSP1*

*NS* not searched

Bold numbers indicated isotig numbers annotated as focal gene

Genes indicated in bold denote the candidate genes identified and annotated in our C. metalifer transcriptome database

**Fig. 4:**
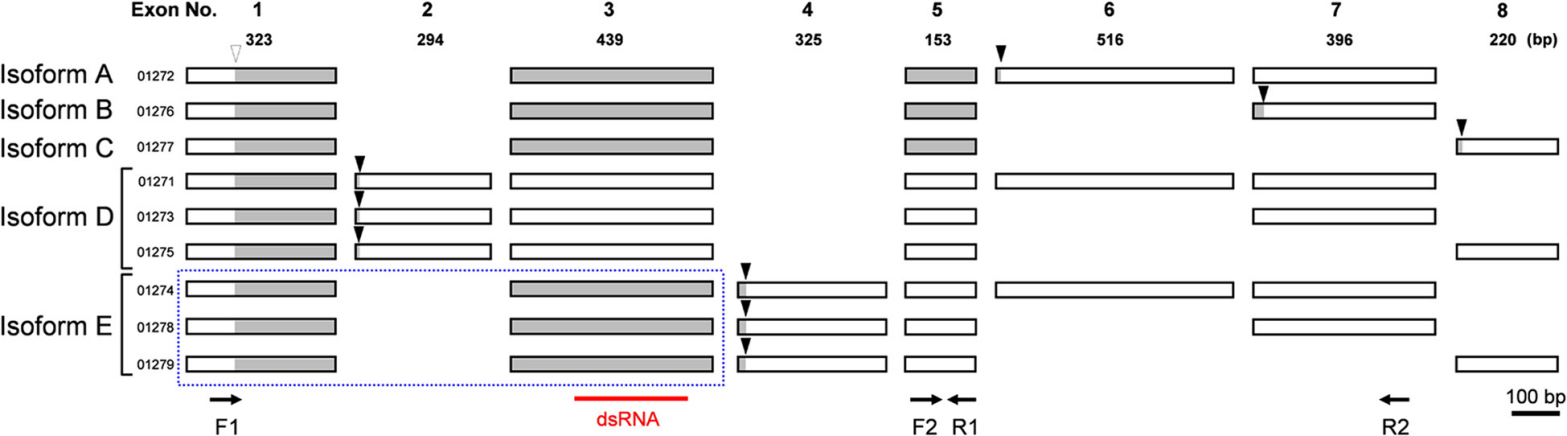
Predicted gene models of the *C. metallifer transformer-2* (*Cmtra2*) gene. Nine different splice variants were predicted from the database assembly and annotation. A total of five different isoforms were predicted (isoform A-E). The coding sequences are in gray, stop codons are indicated by black arrowheads. The open arrowhead indicates the start codon. Arrows indicate forward and reverse primer locations for RT-PCR and the red bar indicates the region for dsRNA synthesis. The blue dashed box indicates the sequences which were identified by 5’ RACE

Notably, the isogroup corresponding to *Cmtra2* contained nine different isotigs, indicating that *Cmtra2* potentially has multiple different splice variants (Fig. 4). Since three splice variants corresponding to isotig 01274, 01278 and 01279 were predicted to lack 5’ sequences, we additionally performed 5’ RACE to identify 5’ sequences of these splice variants. Three of these variants also shared exons 1 and 3 with isotigs 01272, 01276 and 01277. At the protein level, five different putative protein products (isoforms) were encoded (Fig. 4; labeled Isoforms A to E). Isoform A, B and C are similar to each other, that is, they share conserved regions. Differences among the isoforms are only detected in the last several amino acid residues at the 3’ends (Fig. 4). Isoform D and E are shorter than the other three, due to the insertion of stop codons on exon 2 and exon 4. In addition to *Cmtra2*, two isotigs corresponded to the *female lethal d (fl(2)d)* homolog,suggesting the existence of splice variants (data not shown).

**Fig. 5:**
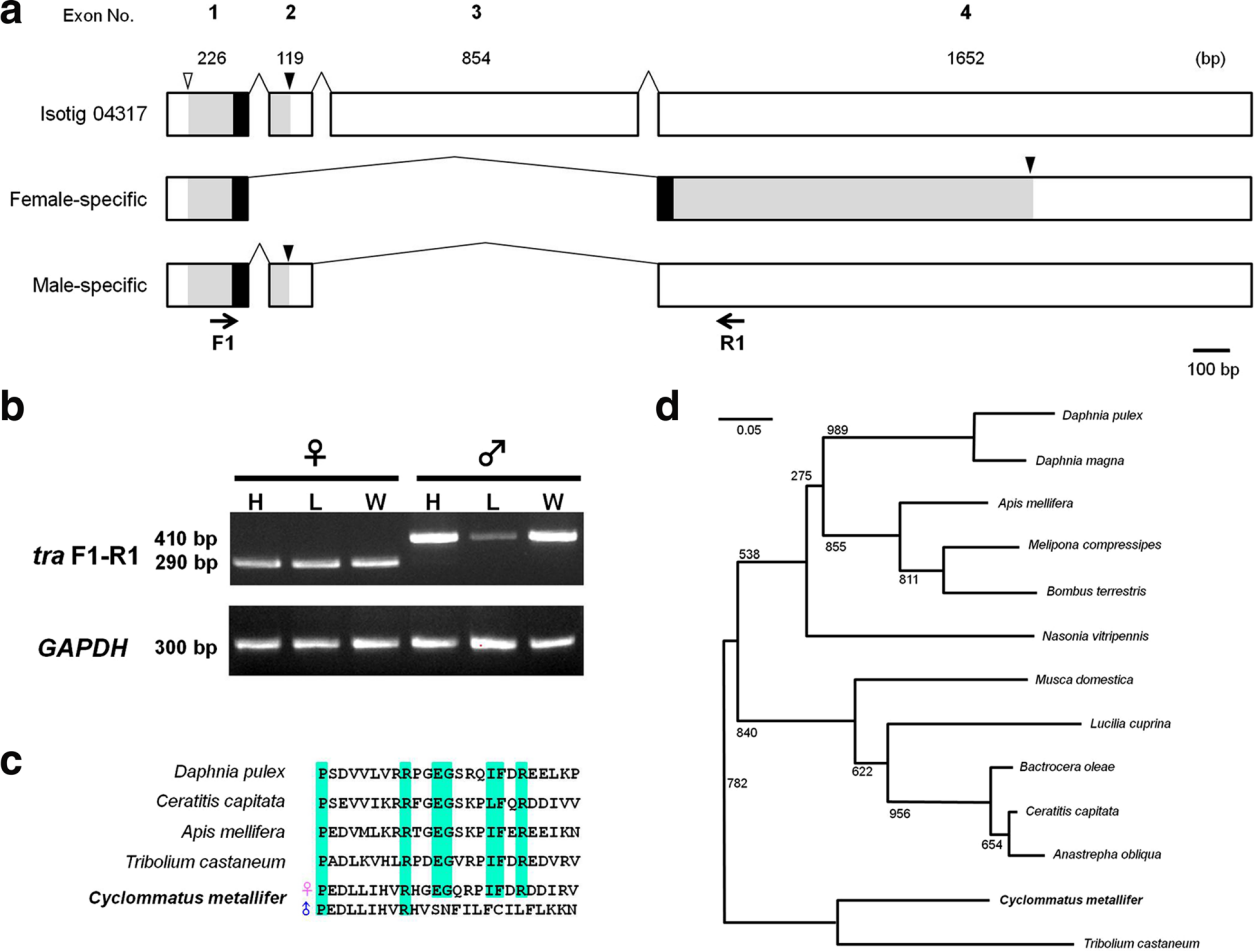
Gene annotation of isotig 04317. **a** Predicted gene model of isotig 04317 (*Cmtra*), and expressed transcripts in females and males. The coding sequences are in gray, stop codons are indicated by black arrowheads. The open arrowhead indicates the start codon. Arrows indicate forward and reverse primer locations for RT-PCR (**b**) Expression patterns of *Cmtra* in females and males. Template cDNAs were derived from Stage 3 prepupae. H: head, L: leg, W: wing. *GAPDH* was used as a positive control. (**c**) Alignment of conserved 25 amino acid sequences of the putative auto regulation domain [37] of the Tra protein. **d** NJ tree of Tra protein from the conserved 25 amino acid auto-regulation domain

Additionally, we found *tra*-like sequence in our database (isotig 04317) by using the *Tribolium tra* homolog as a query, although the homology was relatively low (e-value = 2e^−8^). Expression analyses and subsequent sequencing revealed that gene transcripts and the corresponding putative protein products were different between sexes (Fig. 5a, b). The male-specific isoform was shorter than the female-specific isoform due to an insertion of a stop codon in exon 2 (Fig. 5a). The female-specific isoform possessed a putative auto-regulation domain, which is contained in all *tra* genes identified in other insects except *Drosophila* [37]. This auto-regulation domain was only partially conserved in the male-specific isoform (Fig. 5a, c). Phylogenetic analyses identifying female Tra proteins in other insects indicated that this Tra-like putative protein was grouped with *Tribolium* Tra (Fig. 5d). Thus, we concluded that the *tra*-like isotig 04317 is the *Cyclommatus tra* homolog and named it *Cmtra*.

### Expression analyses of *CmSxl, Cmix,* and *Cmtra2*

**Fig. 6:**
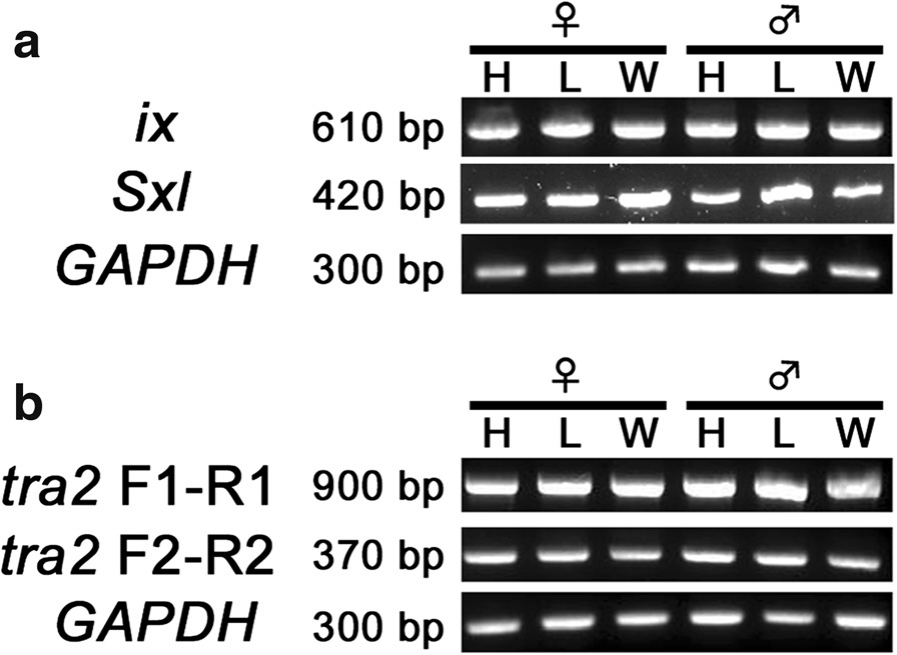
Expression patterns of *CmSxl*, *Cmix* and *Cmtra2.* Template cDNAs were derived from Stage 3 prepupae. H: head, L: leg, W: wing. **a** Expression patterns of *Cmix* and *CmSxl*. Only a single product was amplified regardless of sex or tissue type. **b** Expression patterns of *Cmtra2* using different pairs of primers. See Fig. 5 for their position. Only a single product was amplified regardless of sex or tissue type. *GAPDH* was used as positive control for both analyses

From the 14 candidate *C. metallifer* sex determination genes (Table 2), we selected *CmSxl, Cmix,* and *Cmtra2* for expression and functional analyses because these three genes have well known functions in the sex determination cascade in *Drosophila*. In order to describe the expression patterns of those candidate genes, we conducted semi-quantitative RT-PCR between the sexes and across body parts (head, legs, wings) during the prepupal period. Our results showed uniform expression of *CmSxl* between sexes and among body parts (Fig. 6a). The expression pattern of *Cmix* was also neither sex nor body-parts specific (Fig. 6a). Since there were several assembled isotigs homologous to *Cmtra2* indicating existence of multiple splice variants, we used multiple primers to investigate the expression of specific *Cmtra2* splice variants. Only a single product was detected by RT-PCR using the F1-R1 primer set (Fig. 6b). According to its size, this amplified product is probably composed of exon 1, 3 and 5. Thus, isotig 01272 (isoform A), 01276 (isoform B) and 01277 (isoform C) appear to be the most commonly expressed forms. Products of the isotigs corresponding to isoform D and E were not amplified to a detectable level. In addition, RT-PCR using F2-R2 primers also amplified only a single product with 370 bp predicted size (Fig. 6b), suggesting that isotig 01276 (corresponding to isoform B) had higher expression than isotig 01272 (corresponding to isoform A), and that the major expressed variants are isotig 01276 and/or 01277. We could not find any evidence of sex specific or organ specific expression of *Cmtra2,* regardless of the primer sets used for RT-PCR.

### Functional analyses of *Sxl*, *ix* and *tra2*

**Fig. 7:**
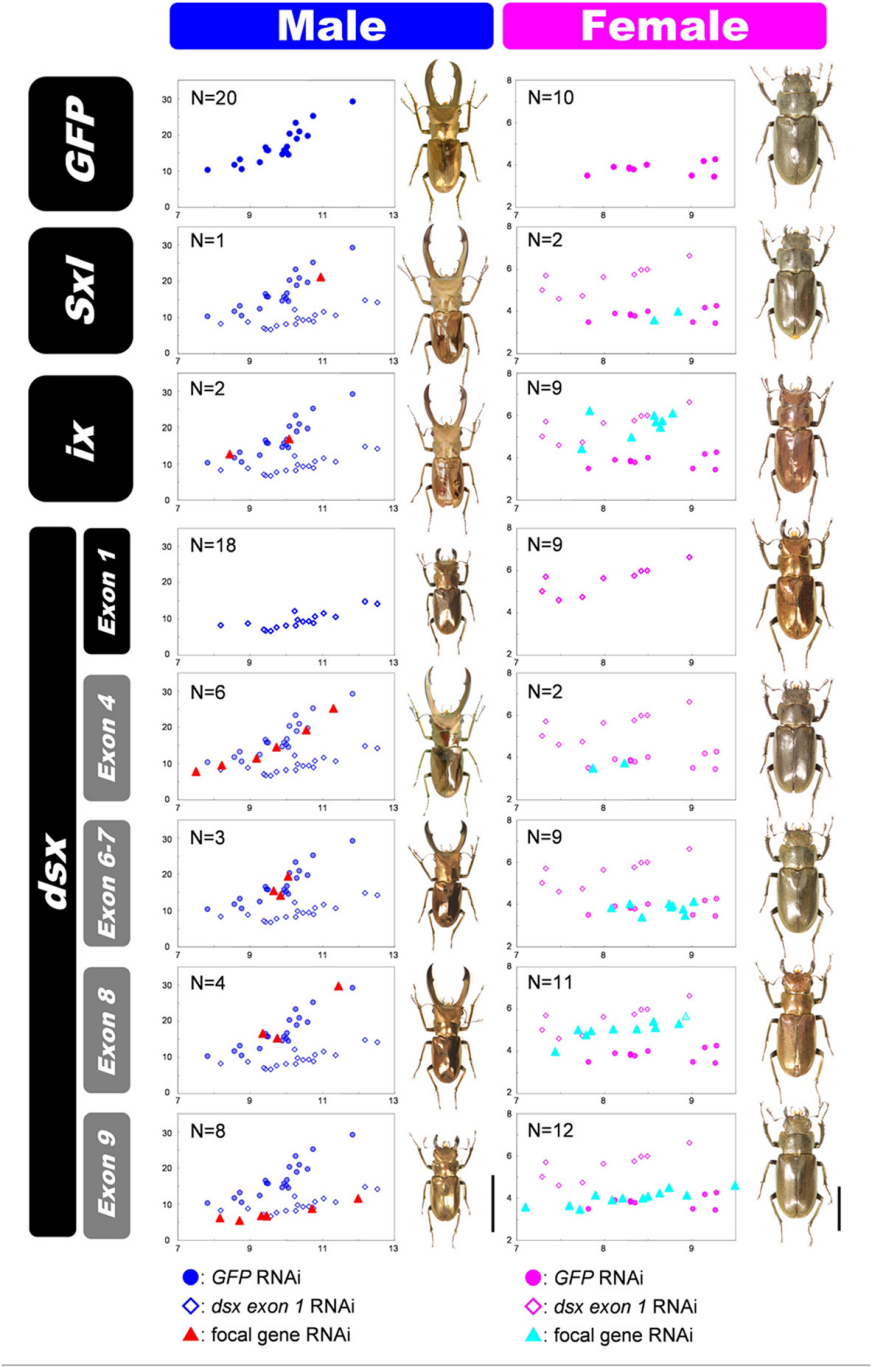
RNAi phenotypes of *Cmdsx* isoforms, *Cmix* and *CmSxl*. Each scatter plot panel indicates the scaling relationship between prothorax width (X-axis) and mandible length (Y-axis). *GFP* ^RNAi^ control individuals (closed circles, male: blue, female: pink) and *Cmdsx-exon 1* ^RNAi^ (open diamonds, male: blue, female: pink) are indicated in all panels. Focal gene/exon RNAi individuals are indicated in closed triangles (male: red, female: light blue). The name of the focal genes or exons for each panel are indicated in the left row. N indicates the sample size for each gene/exon RNAi. Adult phenotypes of each gene knockdown are indicated on the right of each scatter plot panel. Scale bars indicate 20 mm for males and 10 mm for females

We performed gene knockdowns via RNAi during the prepupal period, which is the critical period of sex-specific mandibular tissue proliferation in the stag beetle [31, 39]. Knockdown of *Cmtra2* resulted in death in both sexes (0/27 females and 0/5 males survived); all of the dsRNA injected larvae died during the prepupal period. Knockdown of *CmSxl* did not show any detectable abnormalities in morphology in either sex (Fig. 7). In contrast to these two early acting genes, knockdown of *Cmix* significantly affected sex-specific morphological characters such as mandible size and body color in females (Fig. 7), as predicted from its role in terminal differentiation in *Drosophila*. Specifically, mandible growth was induced in *Cmix*^RNAi^ females compared with control *GFP*^RNAi^ females (Fig. 7). Those *Cmix*^RNAi^ female phenotypes exhibited phenotypes identical to the *Cmdsx*^RNAi^ female phenotypes across all affected traits (Fig. 7). Also, dark non-metallic brown female body color was changed to be more male-like golden metallic color in *Cmix*^RNAi^ females (Fig. 7). In contrast, *Cmix*^RNAi^ males did not show any detectable effects (Fig. 7).

### Identification of undescribed *dsx* variants by RACE

**Fig. 8:**
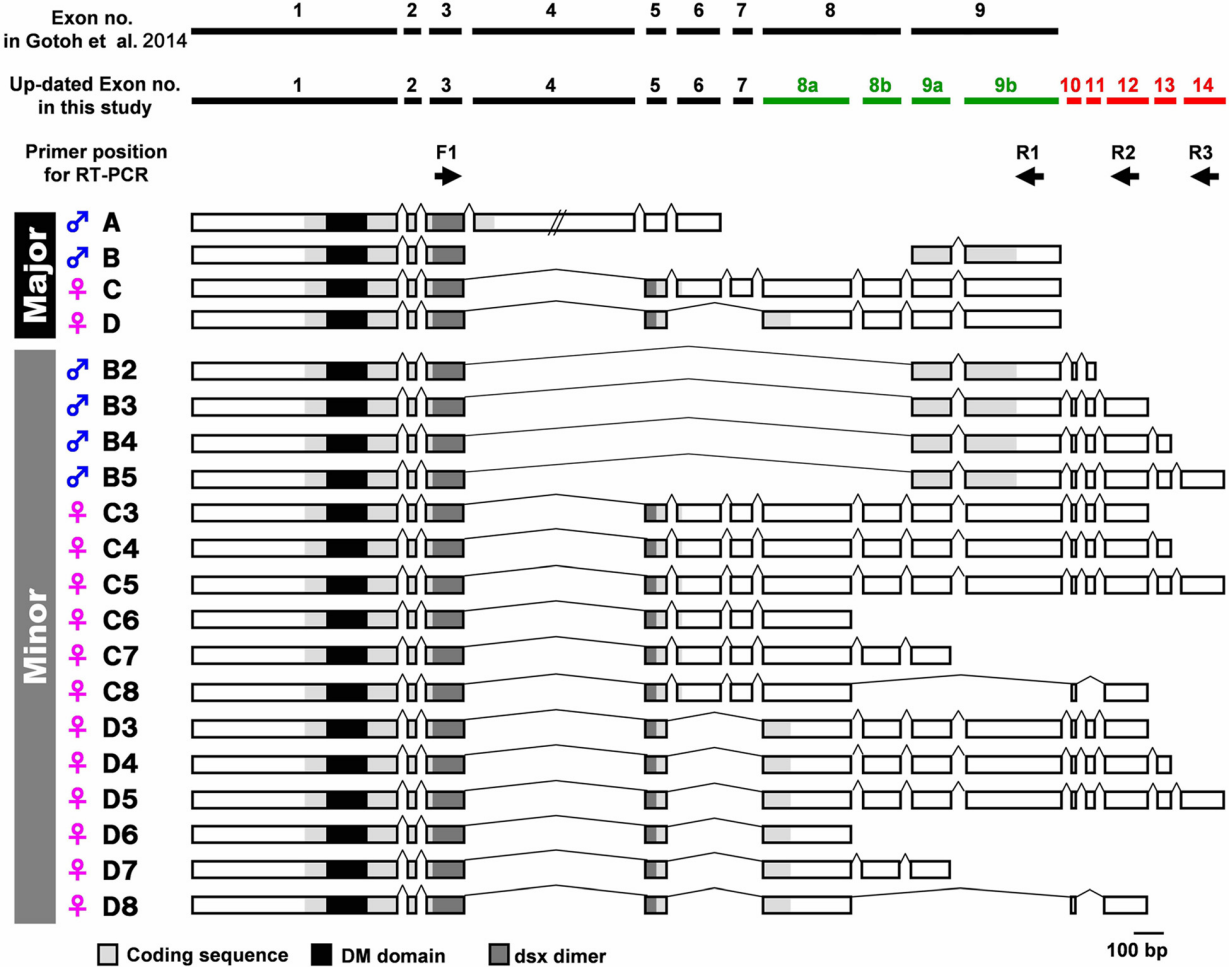
Predicted gene models for *Cmdsx* gene. In addition to the previously reported four splice variants (corresponding to isoforms A to D), we identified 16 new splice variants of *dsx* in *Cyclommatus metallifer*. We updated the exon number from those reported in Gotoh et al. (2014). Exon 8 and exon 9 were divided into 8a, 8b and 9a, 9b, respectively (green bars and numbers). Also, five new exons were named (exon 10 to exon 14; red bars and numbers). Coding sequences are indicated in gray, arrows indicate forward and reverse primer locations for RT-PCR

**Fig. 9:**
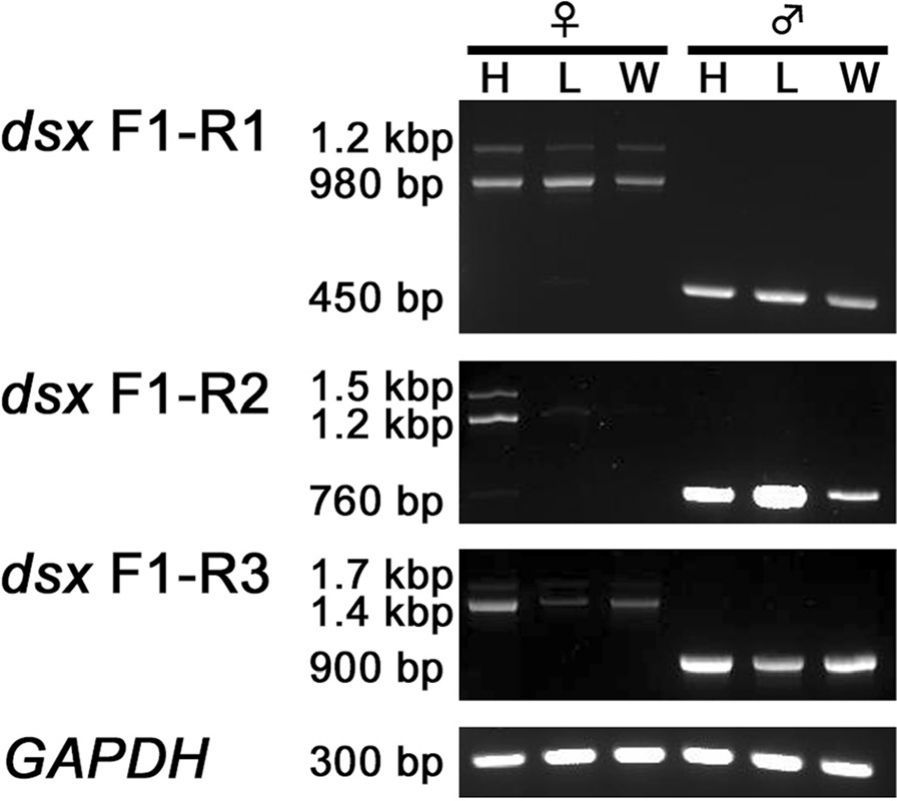
Expression patterns of *Cmdsx* using different pairs of primers. Template cDNAs were derived from Stage 3 prepupae. H: head, L: leg, W: wing. See Fig. 8 for primer positions. Clear differential usages of *Cmdsx* variants between sexes are recognized. *GAPDH* was used as positive control for analysis

Although we were unable to locate a *dsx* homolog sequence in our database, we identified *Cmdsx* sequences by degenerate PCR and subsequent RACE-PCR in a previous study [31]. In Gotoh et al. (2014), we described the sex specific expression pattern and function of four *Cmdsx* splice variants [31]. However, other recent reports of *dsx* genes from other beetle species identified a larger number of *dsx* splice variants than we reported (i.e. - seven in *Onthophagus taurus* [28]; and 11 in *Trypoxylus dichotomus* [30]), thus we tried to clone potential unidentified *Cmdsx* splice variants by RACE-PCR. We identified 16 additional splice variants (Fig. 8). We discovered five new exons at the 3’ end of our previously described variants. These five exons have variation in their combination, which leads to variation in the 3’ UTR among the splice variants (Fig. 8). Our new sequence information also caused us to divide and rename exons 8 and 9 to exon 8a, 8b and exon 9a, 9b. In females, the specific variants of *Cmdsx* C6, C8, D6, and D8 lack exon 8b, 9a and 9b, and variants C7 and D7 lack exon 9b. All of the variants including newly identified exons appeared as faint minor bands during RACE-PCR, while previously identified variants appeared as strong, clear bands (data not shown). Although we found these variations in 3’ UTR length among our 16 splice variants, the number of putative protein products (or the number of CmDsx isoforms) is still four (Fig. 8). We performed semi-quantitative RT-PCR using exon-specific primers (primer positions are indicated in Fig. 8) to get a profile of expression patterns for these *Cmdsx* splice variants during development. RT-PCR expression analyses showed that all of exons 9b, 12 and 14 were used in both males and females, although each variant had sex-specific patterns of expression (Fig. 9). Some of variants were not detected by RT-PCR. For example DNA bands corresponding to C8 and D8 were not amplified in RT-PCR using F1-R2 primer pair (Fig. 8), probably due to low expression levels.

### Functional analyses of splice variant specific knockdown of *Cmdsx* on sex-specific growth of mandibles

The developmental function of *Cmdsx* was previously tested using RNAi against a common region (*exon 1*) to knockdown all CmDsx isoforms [31]. In this study, we more precisely determined the specific function of each *Cmdsx* splice variant using exon-specific RNAi. We designed dsRNA against *Cmdsx exon 4*, *exon 6–7*, *exon 8a–8b* (hereafter *exon 8*) and *exon 9a–9b* (hereafter *exon 9*) which enabled us to knock down each isoform specifically.

In males, only *Cmdsx-exon 9* knockdown affected male-specific morphology, including mandible length (Fig. 7). There was no significant difference in the scaling relationship between *Cmdsx-exon 9*^RNAi^ males and *exon 1*^RNAi^ males in mandible size (*P* = 0.661 slope and *P* = 0.057 intercept, ANCOVA; Fig. 7), while *Cmdsx-exon 9*^RNAi^ males had significantly shorter mandibles compared to *GFP*^RNAi^ controls in our observed body size range (*P* = 0.0004, ANCOVA; Fig. 7). Normal development of other male-specific traits such as the tibial spines and body color were also affected by *Cmdsx-exon 9*^RNAi^. In contrast, knockdown of *Cmdsx-exon 4*, *exon 6–7* and *exon 8* did not affect male mandible scaling relationships (P > 0.05 in both slope and intercept for all three treatments against *GFP*^RNAi^; ANCOVA), or tibial spines or body color at detectable levels (Fig. 7). In females, *Cmdsx-exon 4* and *exon 6* knockdown did not affect morphology (Fig. 7). There were no significant differences in mandible size compared with *GFP*^RNAi^ control females (P > 0.05 in both slope and intercept, ANCOVA). In contrast, *Cmdsx-exon 8* knockdown transformed females to an intersex morphology (Fig. 7); mandibles were relatively longer than those of *GFP*^RNAi^ control females (*P* = 0.0244, ANCOVA). Knockdown of *Cmdsx-exon 9* in females also slightly, but significantly, increased mandible length. There was no significant difference for the regression slopes between *GFP* and *Cmdsx-exon 9*^RNAi^ females (*P* = 0.0847, ANCOVA), but the intercepts of these regression lines differed significantly (*P* = 0.0098, ANCOVA).

## Discussion

### Transcriptome assembly and annotation

The stag beetle *C. metallifer* transcriptomic database we have constructed is a valuable new resource for molecular screening and candidate gene identification in comparative studies of insect biology, particularly those involving exaggerated, sexually selected and sexually dimorphic structures. Our transcriptomic database covers more than 50 % of the total genes in the genome of *C. metallifer,* based on the number of genes predicted based on the *Tribolium castaneum* genome (16,404 genes [41]) and other holometabolous insect genomes (approximately 10,000 – 19,000 genes [42]). Accordingly, more than half (14 out of 24 candidates) of the genes in the *Drosophila* sex determination cascade were contained in our assembled database, suggesting that our database is a useful platform for molecular screening and candidate gene identification in this species. In this study, we extracted RNAs from larval and prepupal heads and thoraces of both sexes. Although this time period is critical to organ growth, and was selected to investigate exaggerated mandible growth [31, 39], future studies may wish to focus on other time periods (embryo, larval, pupal and adult period) and other body parts (for example, the whole embryo, whole thorax and abdomen), in order to recover additional genes expressed in this species.

### Insights for the evolution of insect sex-determination mechanisms and development of sexually dimorphic weapons

Expression and functional analyses of three *C. metallifer* genes in the *Drosophila* sex determination pathway revealed both diversifications of function (*CmSxl* and *Cmtra2)* as well as conservation (*Cmix*) at least in postembryonic sexual character development*.* Our results are the first report of expression and functional analysis of the *Sxl* gene in a beetle species. *Sxl* acts as one of the earliest selector genes for sex determination in *Drosophila* [19, 43, 44]. Functional *Drosophila* Sxl is produced only in females, which splice downstream *tra* mRNA to the functional female isoforms [43, 44]. In addition to controlling the sex-specific splicing of *tra* transcripts, Sxl also promotes its own expression, using an auto-regulatory splicing loop, and activates downstream genes such as *tra2* and *dsxF* to establish female identity [45]. Since Sxl also regulates female-specific dosage compensation, loss-of-function mutants of *Sxl* are lethal only in females [34, 46]. However, outside of flies, the function of *Sxl* in other insects is not well known [47]. We found uniform expression and no detectable defect in knockdown animals suggesting that *CmSxl* does not have a significant role in postembryonic sexual character development in male or female *C. metallifer*. These results are consistent with previous expression and functional studies in non-Drosophilid flies and Lepidopteran insects, suggesting that the *Sxl* sex determination function is not a common mechanism among insects [34] and likely evolved in the *Drosophilid* lineage. Although *CmSxl* did not have a phenotype when knocked down early in the prepupal period of *C. metallifer,* to fully understand its function it will be necessary to investigate additional developmental stages, especially in early embryos when the initial sex-determination signal is likely to function.

Knockdown of the RNA binding protein Tra2 had lethal effects in *C. metallifer*, so we could not examine its effects on adult sexually dimorphic traits. In *Drosophila*, Tra2 functions as a co-factor with Tra for splicing *dsx* transcripts to female-specific *dsx* mRNA [35, 48, 49]. When Tra2 is not present, Tra fails to splice *dsx* into the female specific transcript, resulting in a male-like phenotype. This sex-specific splicing function of Tra2 is conserved in other insects such as *Apis mellifera* [50] and *Tribolium castaneum* [51]. Based on these data, we predicted that *Cmtra2*^RNAi^ would transform females to a more male-like phenotype in the stag beetle. However, contrary to our prediction, all of the *Cmtra2*^RNAi^ male and female larvae died during the prepupal periods without molting into pupae. Similar non-sex specific lethality of *tra2* RNAi was also reported from the red flour beetle *Tribolium castaneum* [51] and the honeybee *Apis mellifera* [50]. Shukla and Palli (2013) have suggested that Tra2 and Tra inhibit the formation of the dosage compensation complex in females and Tra2 is necessary for the formation of the dosage compensation complex in males [51]. However, this hypothesis has not been tested. Additional details of the role of *tra2* during development need to be examined to understand the multiple functions of this gene in insects.

In addition to *Cmtra2*, we also identified a putative homolog of *tra* from our transcriptome. In many previously studied insects, including *Tribolium*, *tra* is known as an up-stream regulating factor of *dsx*-mediated sex-specific alternative splicing [37, 52]. Only females produce functional Tra protein, which results in production of the female-type Dsx isoform. The functional Tra protein also splices the *tra* transcript itself. By this positive auto-splicing feedback, female sexuality is fully established [37]. On the other hand, in males, non-functional Tra protein is produced, and Dsx is consequently expressed in the male-specific isoform. Considering that CmTra also has two sex-specific isoforms and only the female-specific isoform has a complete auto-regulation domain, female-specific auto-splicing feedback might be conserved in *Cyclommatus* sex-determination. Further investigations of *Cmtra* function via RNAi and expression analyses of splice patterns of *dsx* and *tra* transcripts in *Cmtra* RNAi knockdown individuals are necessary for full confirmation of this possibility.

Our data support the conserved function of the Ix protein as a co-factor with female-specific Dsx protein [36, 53]. In *Drosophila* females, *ix* mutant phenotypes are highly similar to *dsx* mutants, while in males, *ix* mutants do not show any detectable morphological defects [53]. Knockdown of the *ix* gene in the milkweed bug *Oncopeltus fasciatus* affected both male and female genital morphological structures [54]. In *C. metallifer*, *Cmix*^RNAi^ affected sex specific trait expression in females only. Interestingly, female *Cmix*^RNAi^ external morphological phenotypes were not detectably different from *Cmdsx*^RNAi^ phenotypes. Based on this, we suggest that CmIx acts together with CmDsxF to specify female differentiation. Although we cannot detect any morphological differences between *Cmix*^RNAi^ and wild type males, we did not evaluate the reproductive abilities of these knockdown animals. RT-PCR indicated that *Cmix* is expressed in male tissues, suggesting that it may have some function in males that we did not detect. Further analyses on the non-morphological traits such as fertility of males, as well as perturbations at additional developmental periods (e.g. early embryos), will be needed to more fully characterize the role of *Cmix* in males.

### Sexual differentiation is regulated by sex-specific expression of Doublesex isoforms

We identified additional splice variants of the *Cmdsx* gene. All of the new transcripts are predicted to encode one of the four previously described isoforms (CmDsxA, CmDsxB, CmDsxC or CmDsxD; [31]). In males, neither *Cmdsx*-*exon 4*^RNAi^ nor *exon 6-7*^RNAi^, which are predicted to knockdown the male biased isoform CmDsxA, affected male morphology. These results suggest that CmDsxA may not have a significant role in male differentiation even though this transcript has a male-biased expression pattern [31]. In females, *Cmdsx*-*exon 6-7*^RNAi^ did not cause any detectable phenotypes although it is predicted to be included in female specific isoform CmDsxC.

In contrast to knockdown of CmDsxA and CmDsxC, knockdown of CmDsxB and CmDsxD did influence sex-specific morphogenesis in males and females. In males, *Cmdsx*-*exon 9*^RNAi^ (predicted to knockdown male specific isoform CmDsxB) resulted in a more female-like phenotype (Fig. 7). These results suggest that CmDsxB is essential for male differentiation. The amino acid sequence of CmDsxB also has high similarity to male-specific Dsx proteins from other beetles [28, 30]. In females, *C. metallifer* CmDsxD specific knockdown (*Cmdsx*-*exon 8*^RNAi^) resulted in a more male-like phenotype (Fig. 7). Thus, in the stag beetle, only CmDsxB and CmDsxD appear critical for generating sex specific morphologies during male and female differentiation. These results indicate a redundancy of CmDsx isoforms in postembryonic sexual differentiation in this species.

Redundancy of Dsx isoforms has also been reported in other beetle species. For example, in the Asian rhinoceros beetle *T. dichotomus*, two different female specific isoforms of Dsx (DsxF-Short and DsxF-Long) have been reported [30]. However, the *T. dichotomus* DsxF-Short specific knockdown did not cause any detectable deformation phenotype in either sex [30]. Also, Kijimoto et al. (2012) reported that *Onthophagus taurus* has several Dsx isoforms and knockdown of one of them (OtDsxb) did not produce any noticeable morphological defects [28]. It is possible that these isoforms are not redundant, but play critical roles in sex determination during embryonic development or in reproductive trait development such as functional spermatogenesis and oocyte development, but these have yet to be measured. Another possibility is that these isoforms have functions in non-morphological sexual characters such as mating behavior. For example, it is known that the sex-determination cascade regulates expression and splice patterns of the gene *fruitless* (*fru*) in *Drosophila* [55]. Coordinated actions of *fru* and *dsx* are necessary for male-specific neural development for adult male sexual behaviors [56, 57]. Future functional analyses *of* CmDsxA and CmDsxC focusing on other non-morphological traits will be needed to understand the *dsx* molecular evolution.

## Conclusion

The transcriptome we generated for the prepupal development period in the stag beetle is a useful and important resource for identifying genes involved in morphological evolution including the regulation of sex determination and sexual dimorphism. The ability to identify and functionally screen entire suites of candidate genes, such as those in the sex determination pathway, is a powerful step toward a deeper understanding of both the diversification as well as the conservation of developmental mechanisms in the evolution of complex traits.

## Methods

### Beetle rearing

The golden metallic stag beetle *Cyclommatus metallifer* was purchased from the beetle pet store Hercules-Hercules in Sapporo, Japan. Adults were reared and bred in the laboratory as described previously [39]. Induction of large and small males was also described in Gotoh et al. 2011. Briefly, we kept larvae individually starting from the second instar in 430 mL (large males) or 120 mL (small males) plastic cups filled with decaying wood flakes to induce large and small males.

### Construction of the transcriptome database

Six mRNA libraries were constructed for sequencing from individuals sampled at three stages of larval development (3^rd^ instar larval stage, early pre-pupal stage, and late pre-pupal stage). Libraries at each stage included heads from large males, prothoraces from large males, heads from small males, prothoraces from small males, heads from females, and prothoraces from females. In total, we sampled from 102 individuals evenly spread across the treatments.

Tissues were dissected and stored in RNALater before being transferred to TRiZOL® (Invitrogen, USA) and homogenized using a hand held tissue tearer and left overnight at −80 °C. RNA extraction was then carried out using TRIZoL following the manufacturer’s guidelines, followed by treatment with Ambion® DNA-free™ DNase, before assaying quantity with a NanoDrop (Thermo Scientific, USA) and quality with a BioAnalyzer 2100 (Agilent Inc., USA). For quality control, two aliquots of RNA were taken per extraction, one was left at 37 °C for two hours, while the other was stored at 4 °C. Both aliquots were run on an Agilent RNA nano-chip (Agilent Inc., USA) and the resulting electropherograms inspected for signs of degradation. If any degradation was observed, the extraction was discarded. It should be noted that the RIN value system [58] is not applicable to beetle species since, as in other insect species, the 28S ribosomal peak collapses into the 18S ribosomal peak [59].

Total RNA for each library was then pooled and three rounds of mRNA enrichment were carried out using the Oligotex® mRNA purification kit (Qiagen Inc., USA). Again quantity, quality, and confirmation of substantial mRNA enrichment were performed using a NanoDrop (Thermo Scientific, USA), and quality, with a BioAnalyzer 2100 (Agilent Inc., USA).

Samples were then taken to the Washington State University Molecular Biology Core Laboratory for sequencing library preparation and subsequent sequencing using the 454 GS FLX Titanium Series (Roche, USA), following manufacturer’s protocols. Briefly, mRNA was fragmented using ZnCl_2_ and heat prior to cDNA synthesis. Unique molecular identifiers were ligated to each library and titrated to achieve 8–10 % enrichment and the sequencing beads for each library were made in separate reactions. Sample balance was achieved by loading the picotiter plate with an equal number of enriched beads, determined by coulter counter, for each library.

### Bioinformatics analysis

Prior to analysis, basic statistics on the sequenced reads were obtained through scripts provided by Dr. David Rosenkranz (Johannes Gutenberg University, Mainz). All adapter nucleotides were trimmed and the 454 sequences were *de novo* assembled using the -cdna flag to signify transcriptome assembly in the Newbler 2.9 software package using default parameters (454 Life Sciences/Roche). The resulting isotigs were annotated using a BLASTx search [40] against the GenBank non-redundant database using an e-value threshold of e ^−5^ to identify putative homology. Blast2GO [60, 61] was used to assign GO (Gene Ontology) terms and KEGG (Kyoto Encyclopedia of Genes and Genomes) based metabolic pathways [62, 63]. Final GO assignments were defined based on a 10 % filter for all three processes profiled at level 2. All other settings for the analysis were maintained at their defaults.

### Database search against sex-determination pathway genes

The 24 candidate genes of the sex-determination pathway were selected from the genes categorized as “sex determination (BSID: 492283)” in BioSystems database at NCBI (http://www.ncbi.nlm.nih.gov/biosystems/) [64]. All of those genes were identified in *Drosophila melanogaster*. We searched candidate genes against the database using the tBLASTn algorithm. We used the *Drosophila* and *Tribolium* homologs of those candidate genes (Table 2) as query sequences for the tBLASTn searches. For the *Tribolium* homologs, we used the annotated gene sequences from OrthoDB7 (http://cegg.unige.ch/orthodb7). To further confirm the orthology of detected genes, we made multiple alignments of candidate genes with the orthologs from other insect species, and constructed unrooted trees of mRNA sequences using ClustalX program [65] (http://www.clustal.org/) (Additional file 1: Figure S1-S14). We used the conserved 25 amino acid auto-regulation domain [32] from previously studied insect *tra* genes for constructing the NJ tree for *tra*, using the ClustalX program.

### Identify full-length *Cmdsx* gene by RACE-PCR

Rapid amplification of cDNA ends (RACE) -PCR was performed to obtain the full length of undescribed *Cmdsx* transcripts sequence using the 3’-RACE primer (5’-CTC GAA GAT TGC CAT AAG CTC CTG GAA AGG-3’) and the SMART RACE cDNA Amplification Kit (Clontech, Palp Alto, CA). Also, we performed 5’-RACE for *Cmtra2* and *Cmtra* using 5’-RACE primer (*Cmtra2*: 5’- GCG TAG GCG TGT GAG CCC TCT CTG TG -3’, *Cmtra*: 5’- CCG CAA CTT TGA TGG TTT TGA GTT CCT G -3’). The amplified cDNA fragments were subcloned and sequenced as described in Gotoh et al. (2014).

### Expression analyses of candidate genes using RT-PCR

The expression patterns of our candidate genes in males and females were examined during the prepupal period using RT-PCR. Head, wings and legs were dissected from St-3 prepupae (prepupae which had just undergone the gut purge, see Gotoh et al. 2011 for a detailed definition of the prepupal stages) of each sex. Dissected tissues were preserved at −20 °C in RNA later (Ambion Inc, Austin, TX) until extraction. RNA was extracted from dissected tissues with the GeneJET RNA purification kit (Thermo Fisher Scientific Inc, Waltham, MA) according to the manufacturer’s protocol. For each sample, 2000 ng of total RNA was reverse transcribed with the iScript cDNA Synthesis kit (Bio-Rad, Hercules, CA). These synthesized cDNAs were used as templates for RT-PCR. See Additional file 2: Table S1 for primer sequences. The PCR step was performed under the following conditions: 94 °C for 2 min; 37 (for *Cmix*, *CmSxl* and *Cmtra2*) or 40 (*Cmdsx*) cycles of 94 °C for 30s, 54 °C for 30s and 72 °C for 3 min; and 72 °C for 10 min, using Go Taq Master Mix (Promega, Madison, WI). For *Cmtra*, the PCR program was: 94 °C for 2 min; 40 cycles of 94 °C for 30s, 54 °C for 30s and 72 °C for 4 min; and 72 °C for 10 min, using AmpliTaq360 DNA polymerase (Applied Biosystems). *GAPDH* was used as a positive control.

### Functional analyses of candidate genes via RNAi

Functional analysis of our three target sex-determination genes (*Cmix, CmSxl, Cmtra2*) and each *Cmdsx* splice variant was accomplished with RNA interference (RNAi) during prepupal development. To silence candidate transcripts, double-stranded RNA (dsRNA) against either each target gene (*Cmix, CmSxl, Cmtra2*) or each target exon (*Cmdsx* exon 4, 6–7, 8 and 9) was synthesized. Using primers with the T7 sequence in the 5’-end, partial sequences of target sequences were amplified by PCR. Primer sequences are described in Additional file 3: Table S2. dsRNA synthesis were performed with MEGAscript RNAi Kit (Ambion, Austin, TX) according to the manufacturer’s protocol. For *Cmdsx exon 1* and *GFP,* we used dsRNAs which were used in our previous study on *Cmdsx* function [31]. All dsRNA was diluted with PBS. One μg (which is sufficient to strongly knock down gene expression in *C. metallifer* [31]) of dsRNA in 5 μl of PBS was injected into the dorsal prothorax of late 3rd instar larvae using a microliter syringe (Hamilton, Reno, NV) under a stereomicroscope. Individuals used for subsequent morphological analyses were those that successfully eclosed into adults. Sample sizes are described in Fig. 7. For statistical tests of RNAi effects on mandible size, analysis of covariance (ANCOVA) were performed with body size as a covariate using R 3.0.2 software.

## Additional files

Additional file 1: Figure S1-S14. Constructed unrooted phylogenetic trees of candidate sex-determination genes using ClustalX program. (DOCX 3072 kb) Additional file 2: Table S1. List of primer sequences for RT-PCR. (XLS 18 kb) Additional file 3: Table S2. List of primer sequences for dsRNA synthesis. (XLS 18 kb)
