# Supplementary material for: Identification and functional analyses of sex determination genes in the sexually dimorphic stag beetle Cyclommatus metallifer
Source: BMC Genomics. 2016 Mar 22;17:250. doi: 10.1186/s12864-016-2522-8 (PMC4802893; doi:10.1186/s12864-016-2522-8)
Supplement: Additional file 1: Figure S1-S14. — Constructed unrooted phylogenetic trees of candidate sex-determination genes using ClustalX program. (DOCX 3072 kb) [file 12864_2016_2522_MOESM1_ESM.docx]

Additional file1 of Gotoh et al. 2016, Identification and functional analyses of sex determination genes in the sexually dimorphic stag beetle *Cyclommatus metallifer*

Caption for entire file;

Constructed unrooted phylogenetic trees of candidate sex-determination genes using ClustalX program.


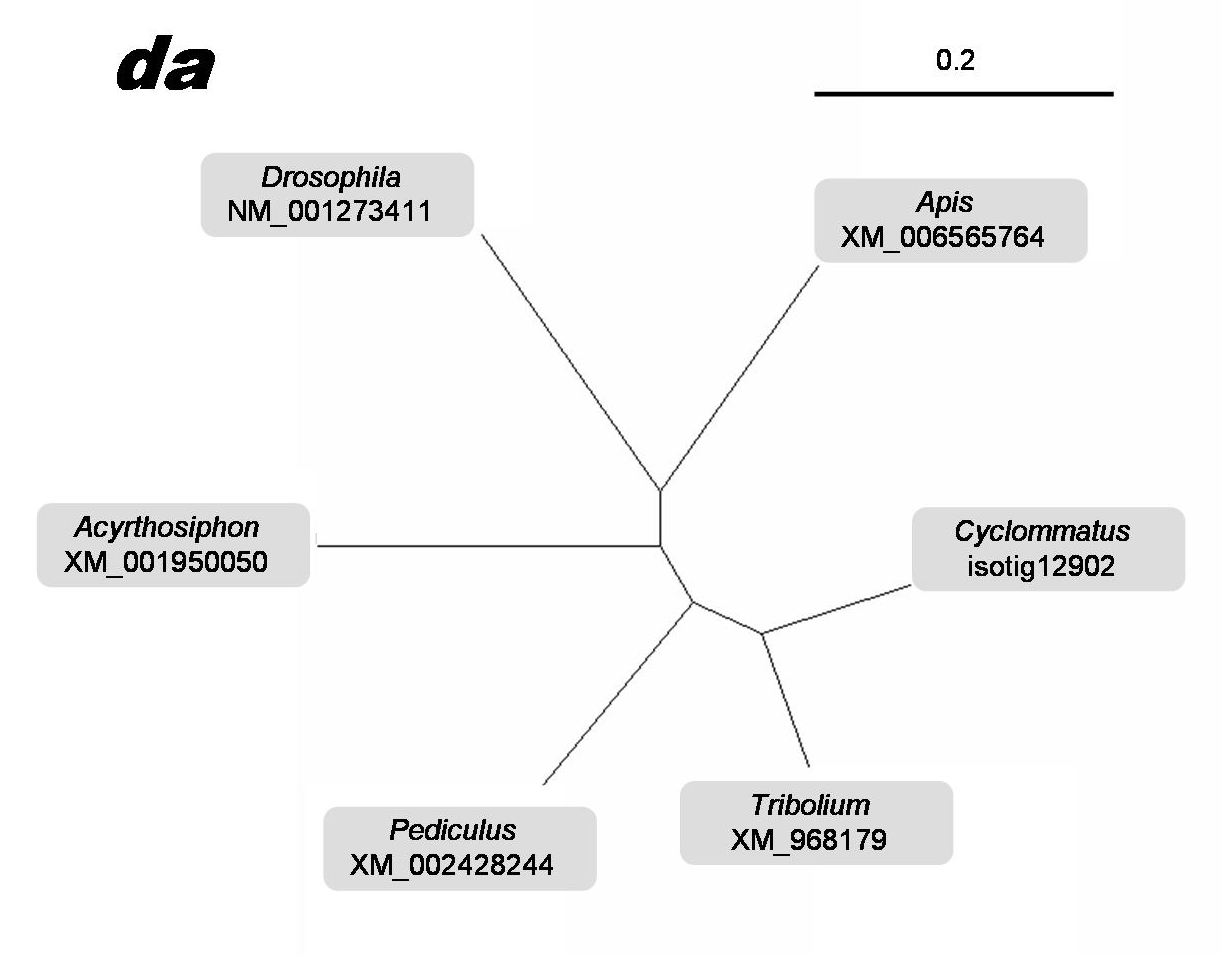


Figure S1. Unrooted phylogenetic tree of *da*.


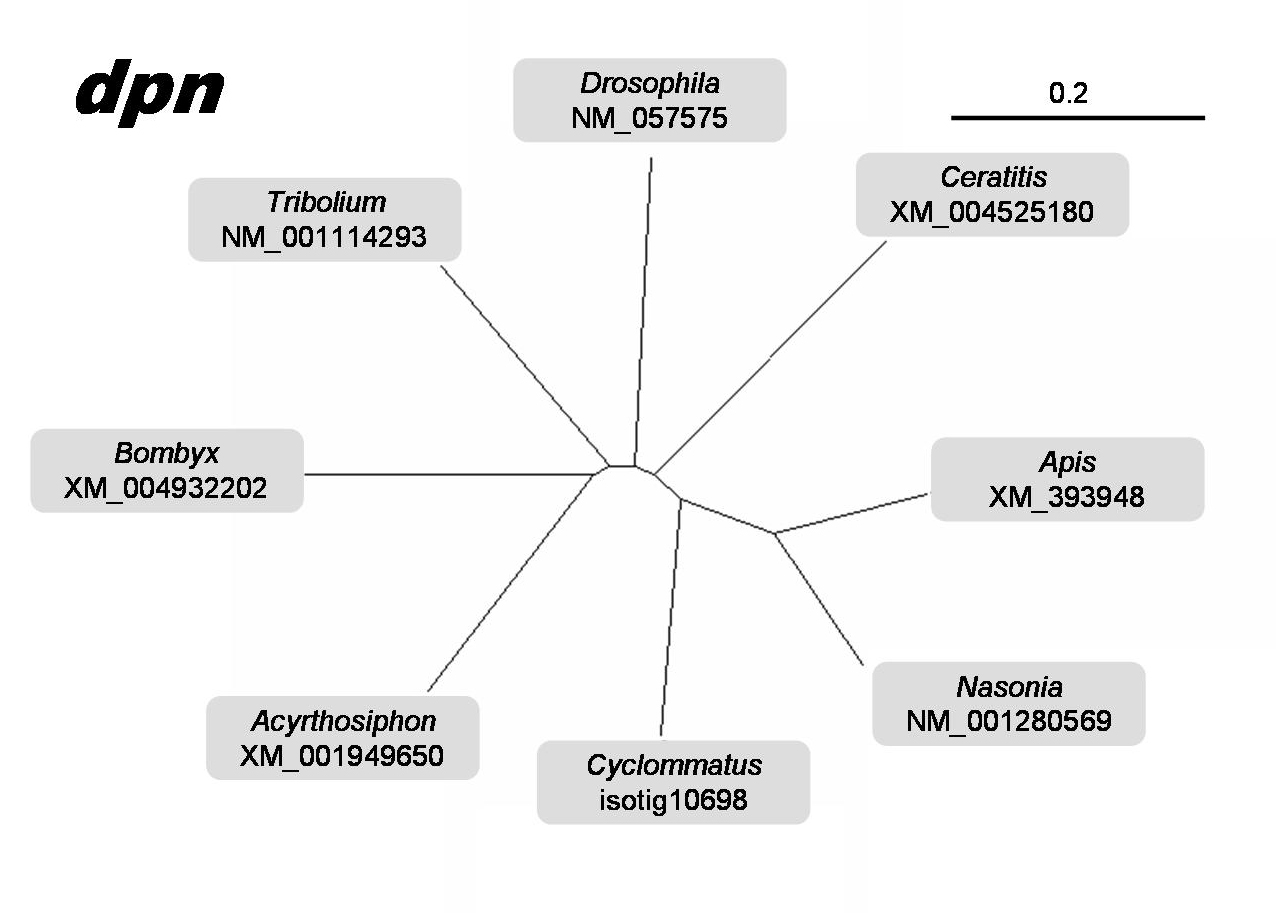


Figure S2. Unrooted phylogenetic tree of *dpn*.


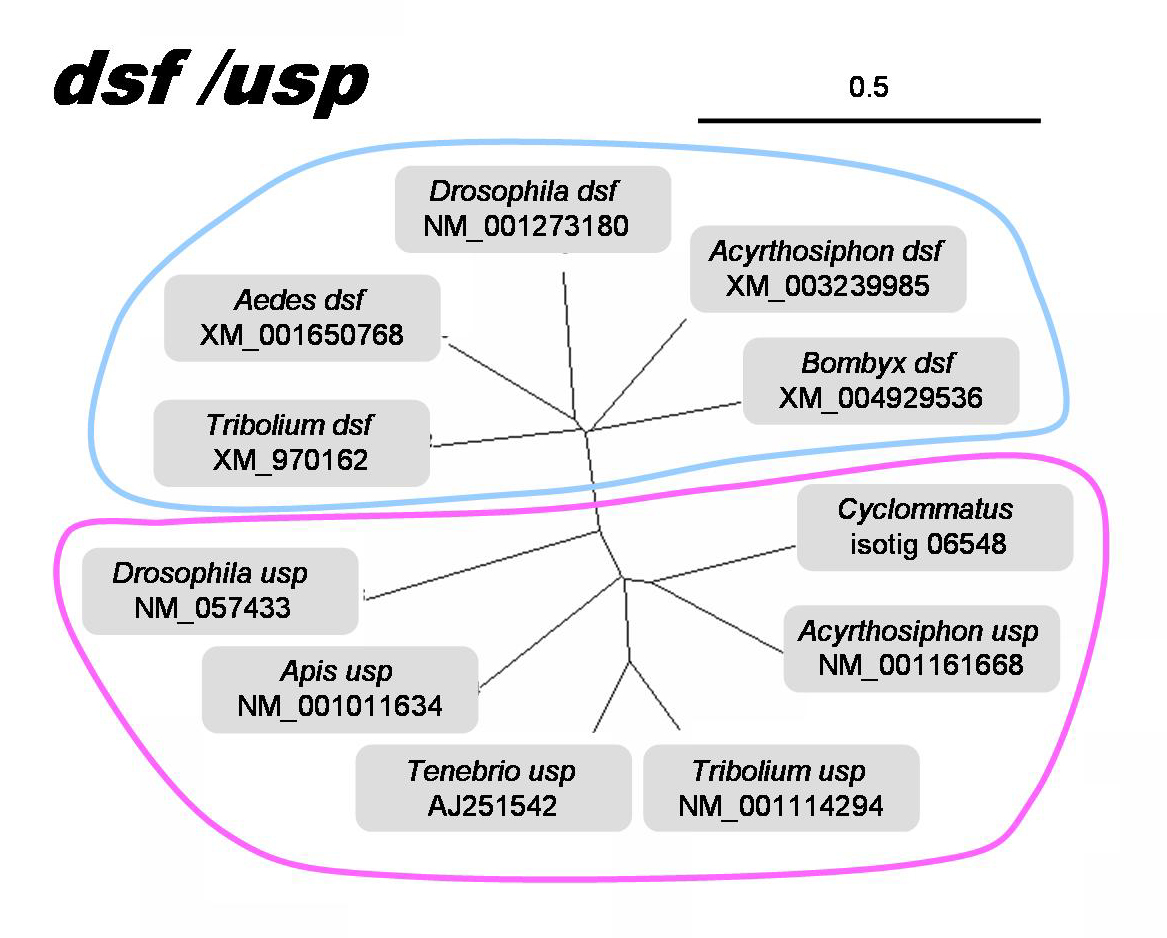


Figure S3. Unrooted phylogenetic tree of *dsf*.


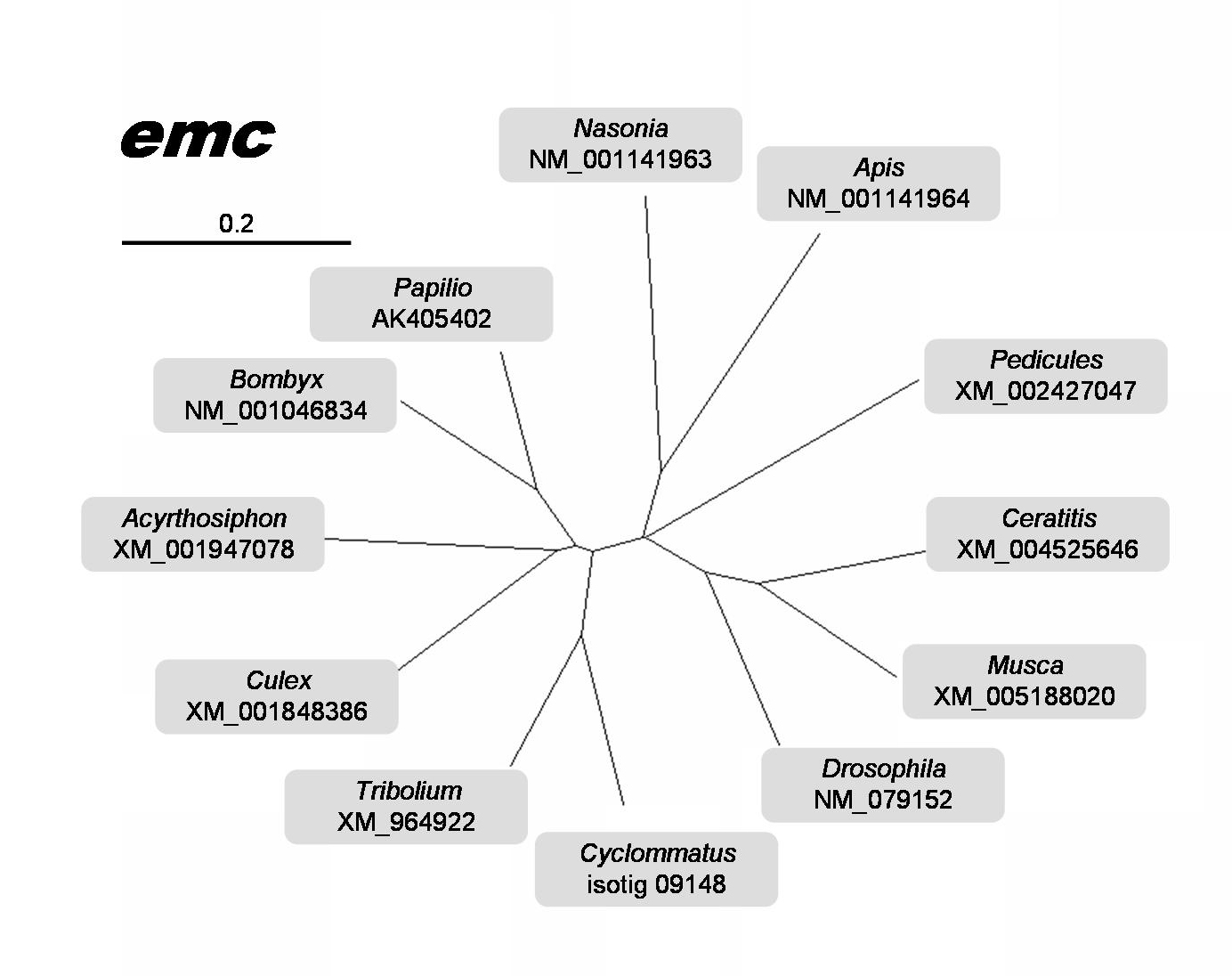


Figure S4. Unrooted phylogenetic tree of *emc*


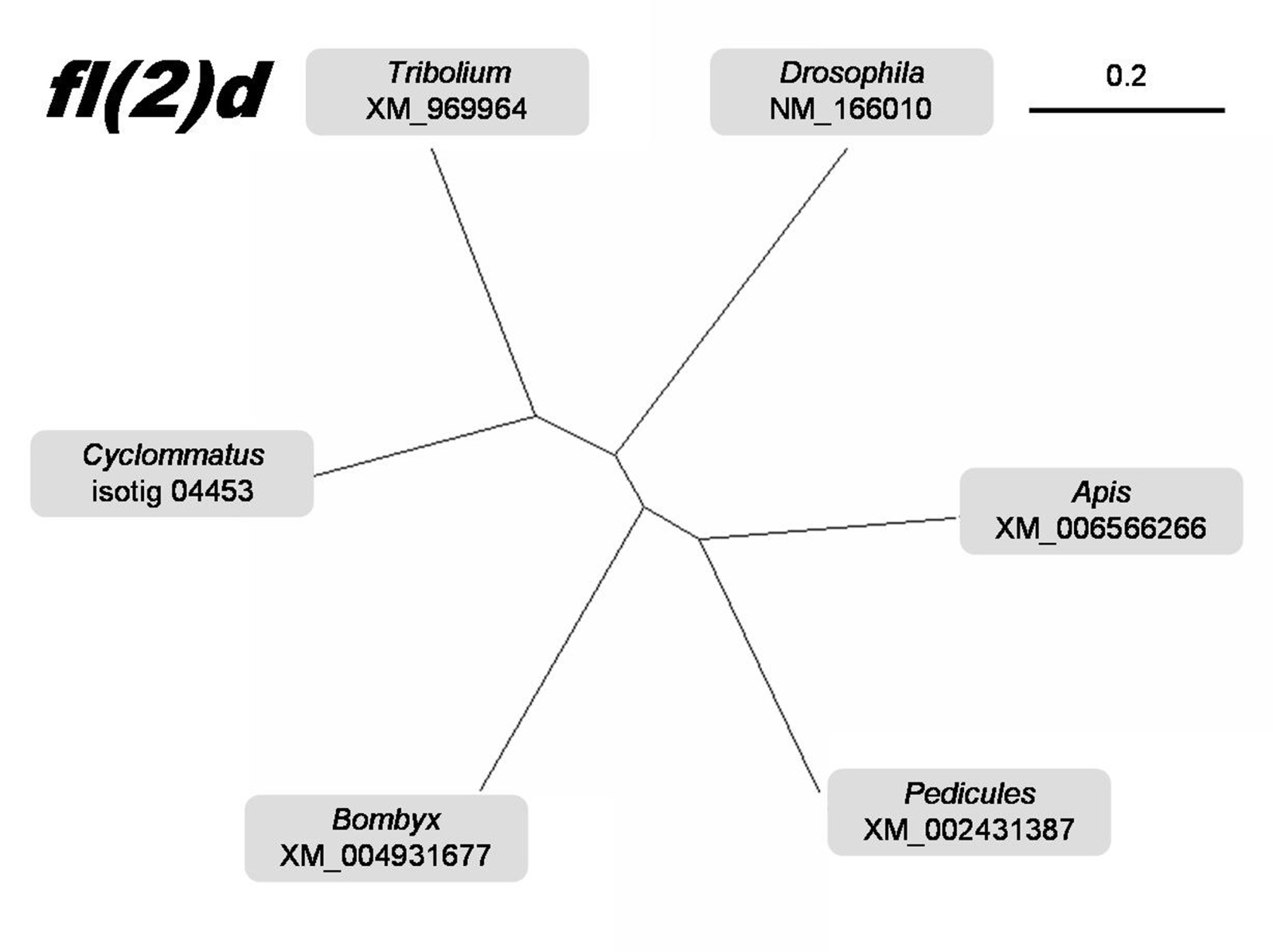


Figure S5. Unrooted phylogenetic tree of *fl(2)d*


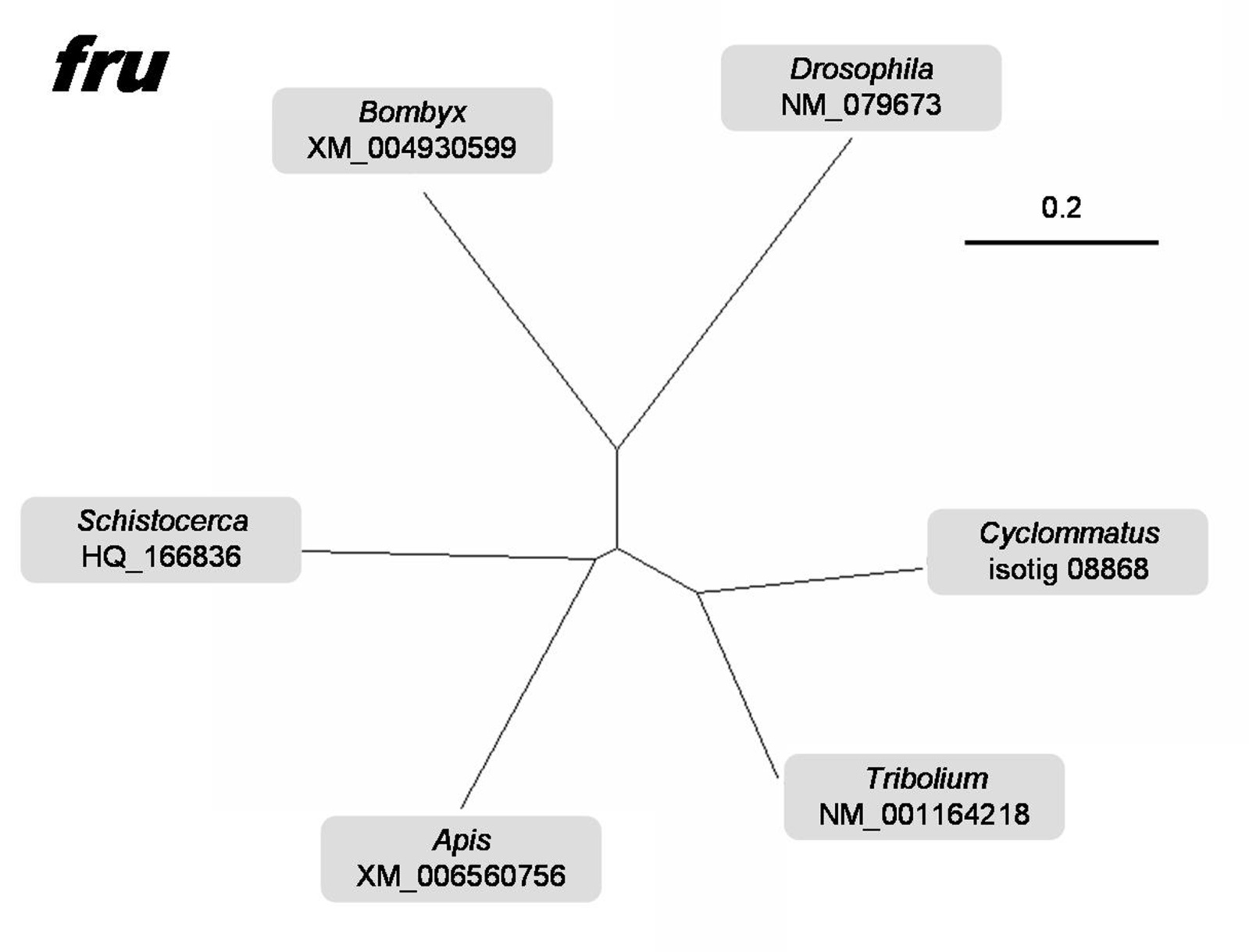


Figure S6. Unrooted phylogenetic tree of *fru*.


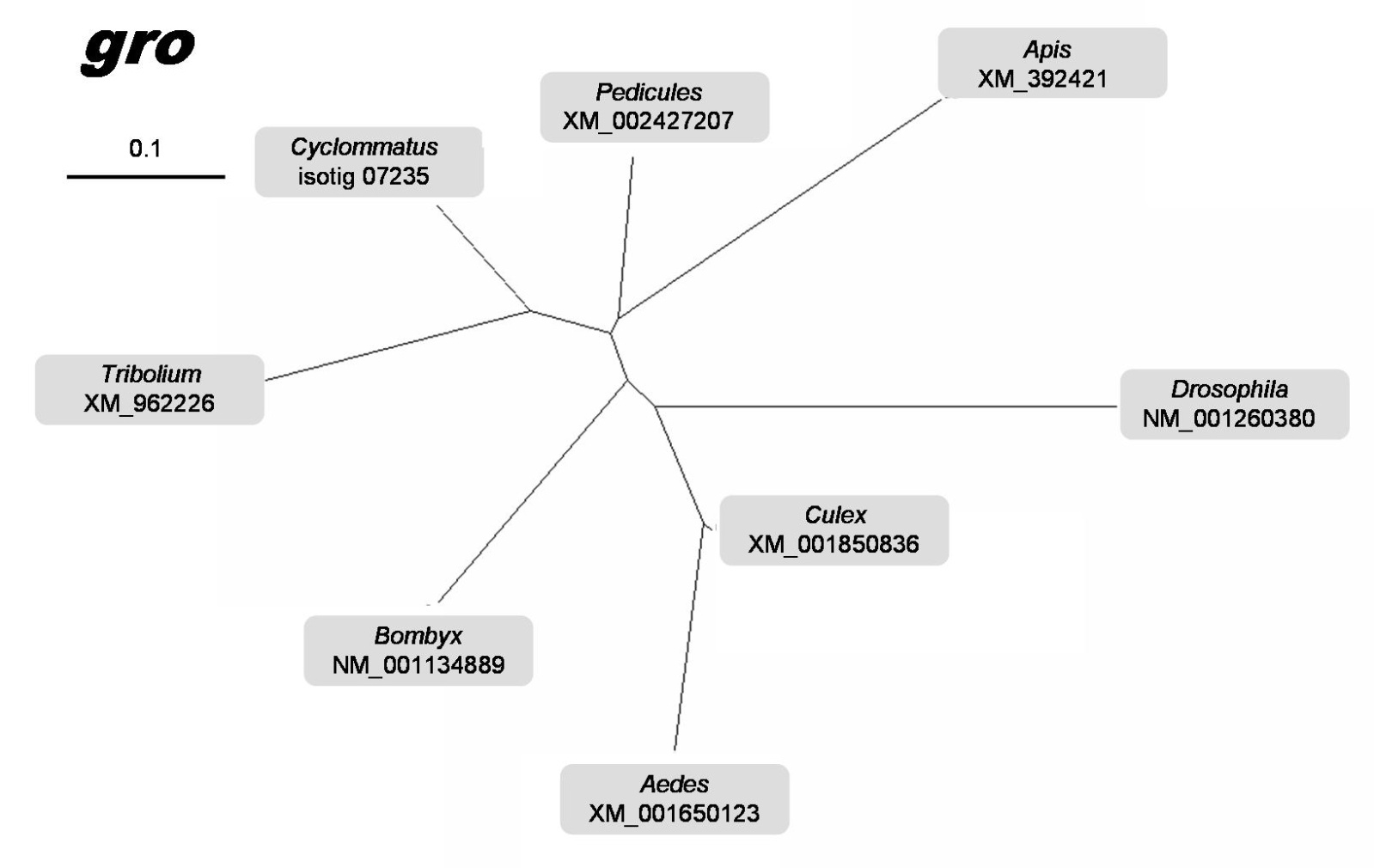


Figure S7. Unrooted phylogenetic tree of *gro*.


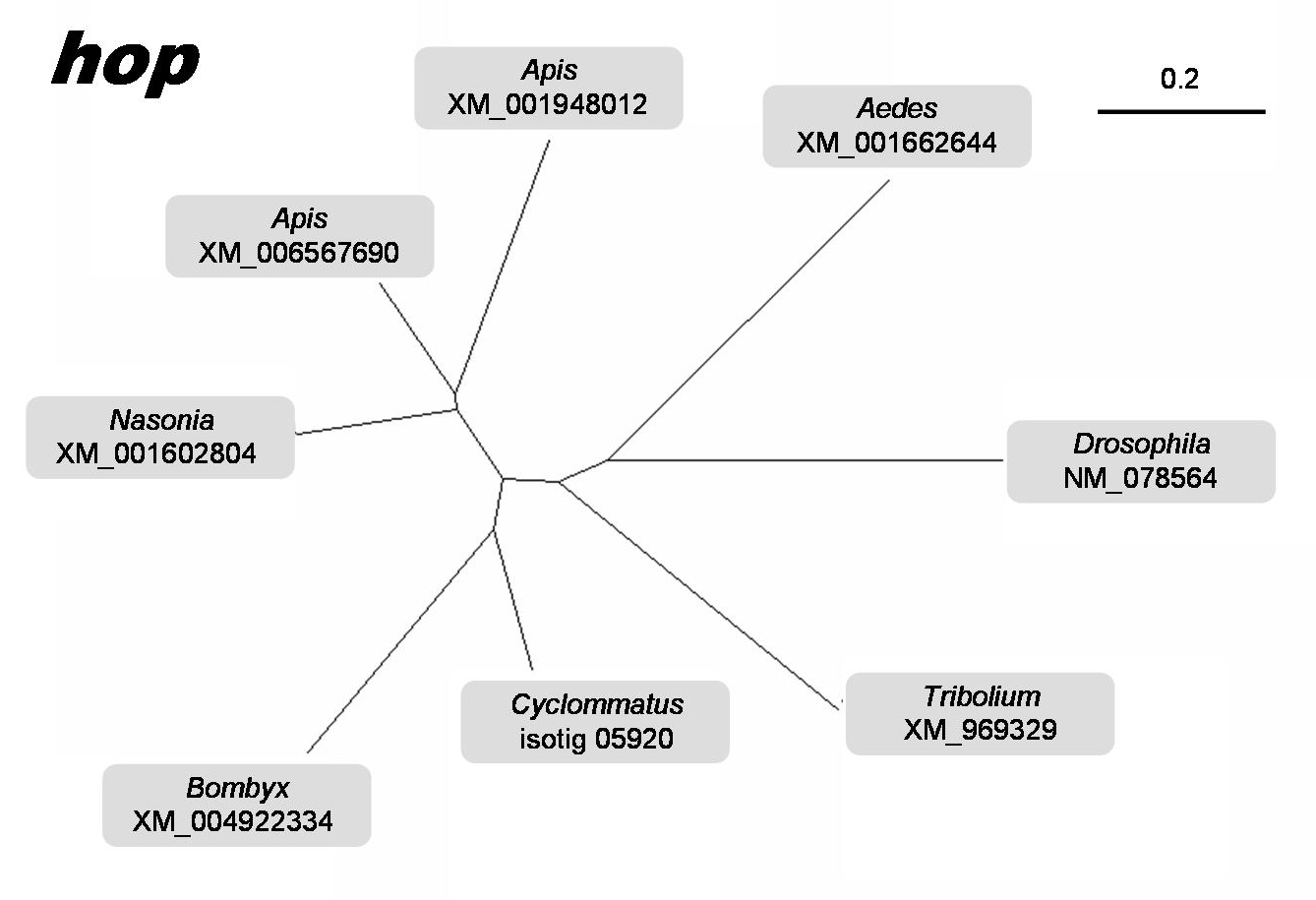


Figure S8. Unrooted phylogenetic tree of *hop*


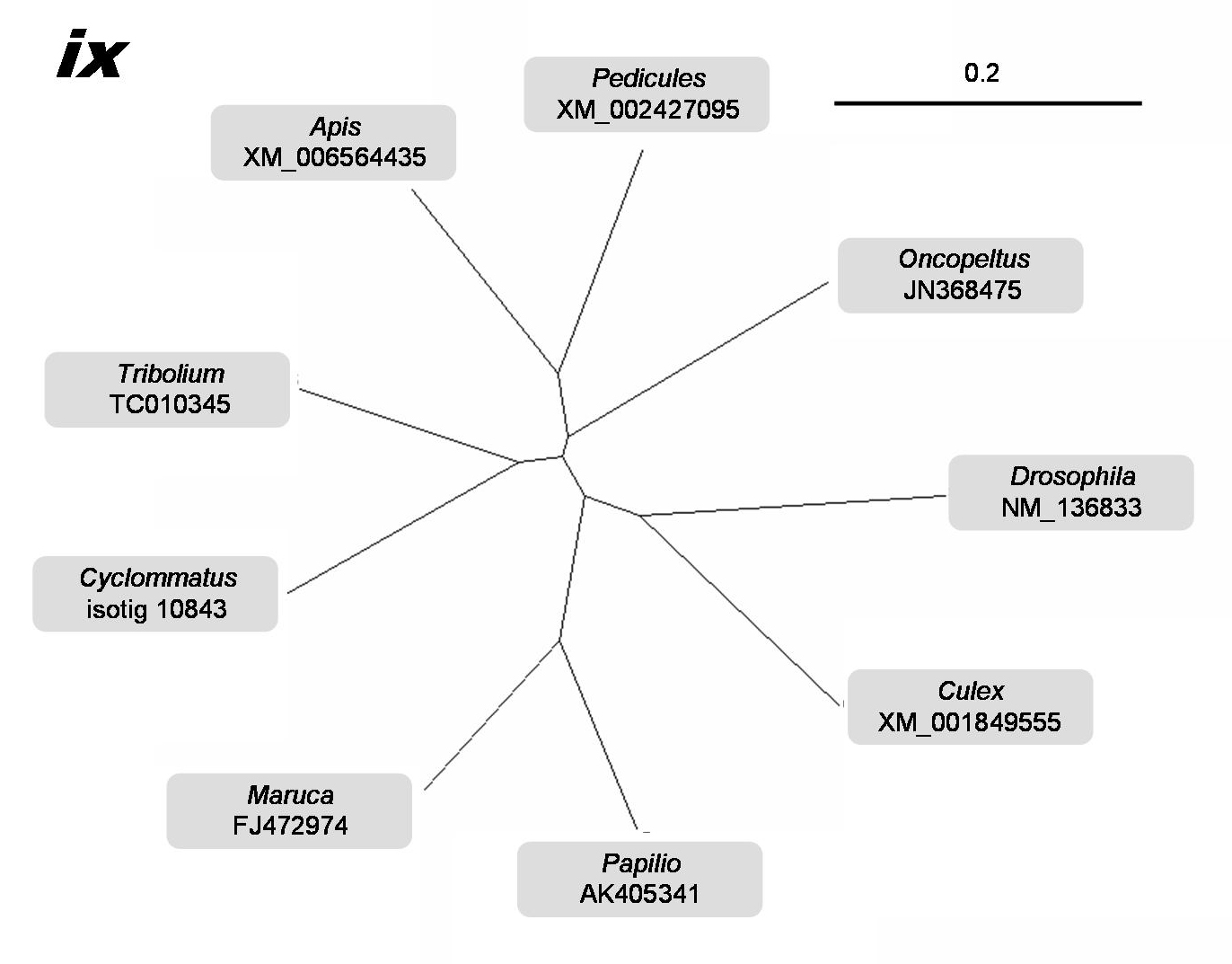


Figure S9. Unrooted phylogenetic tree of *ix*


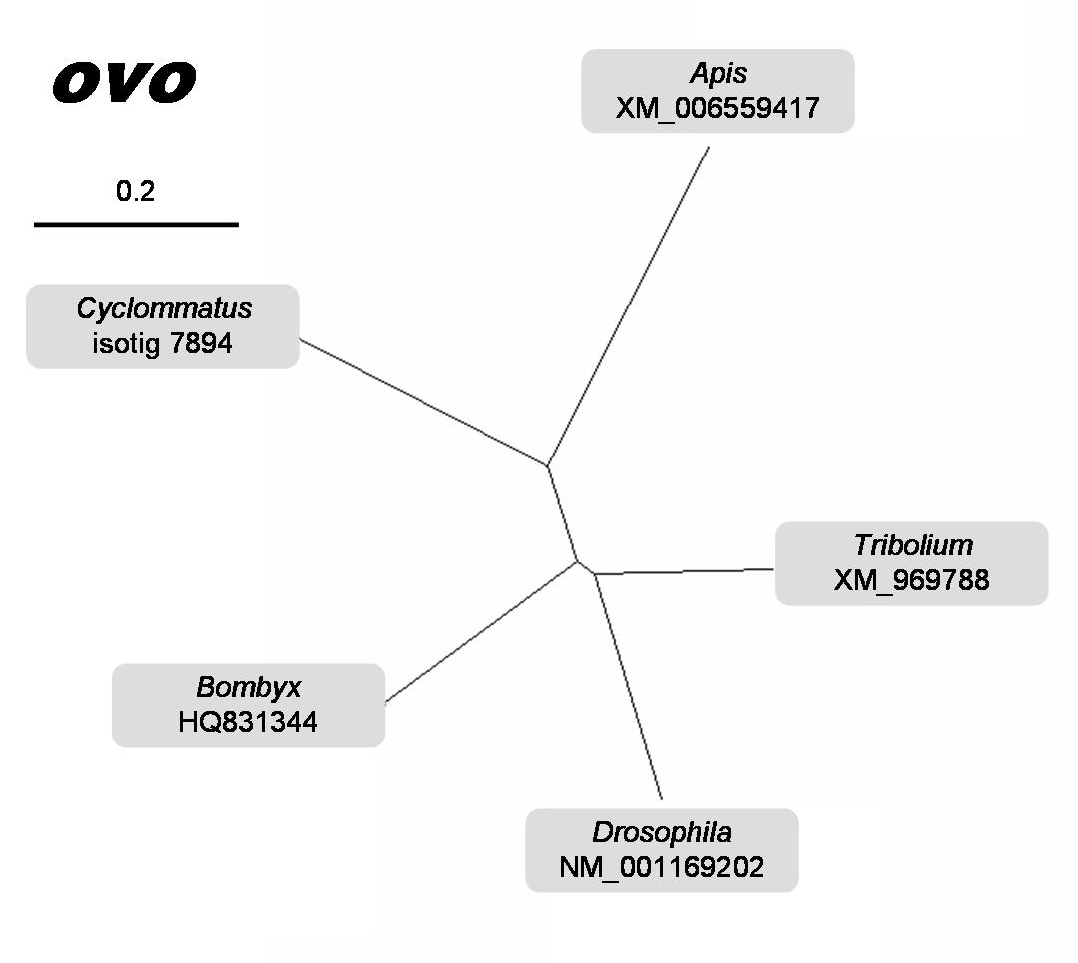


Figure S10. Unrooted phylogenetic tree of *ovo*.


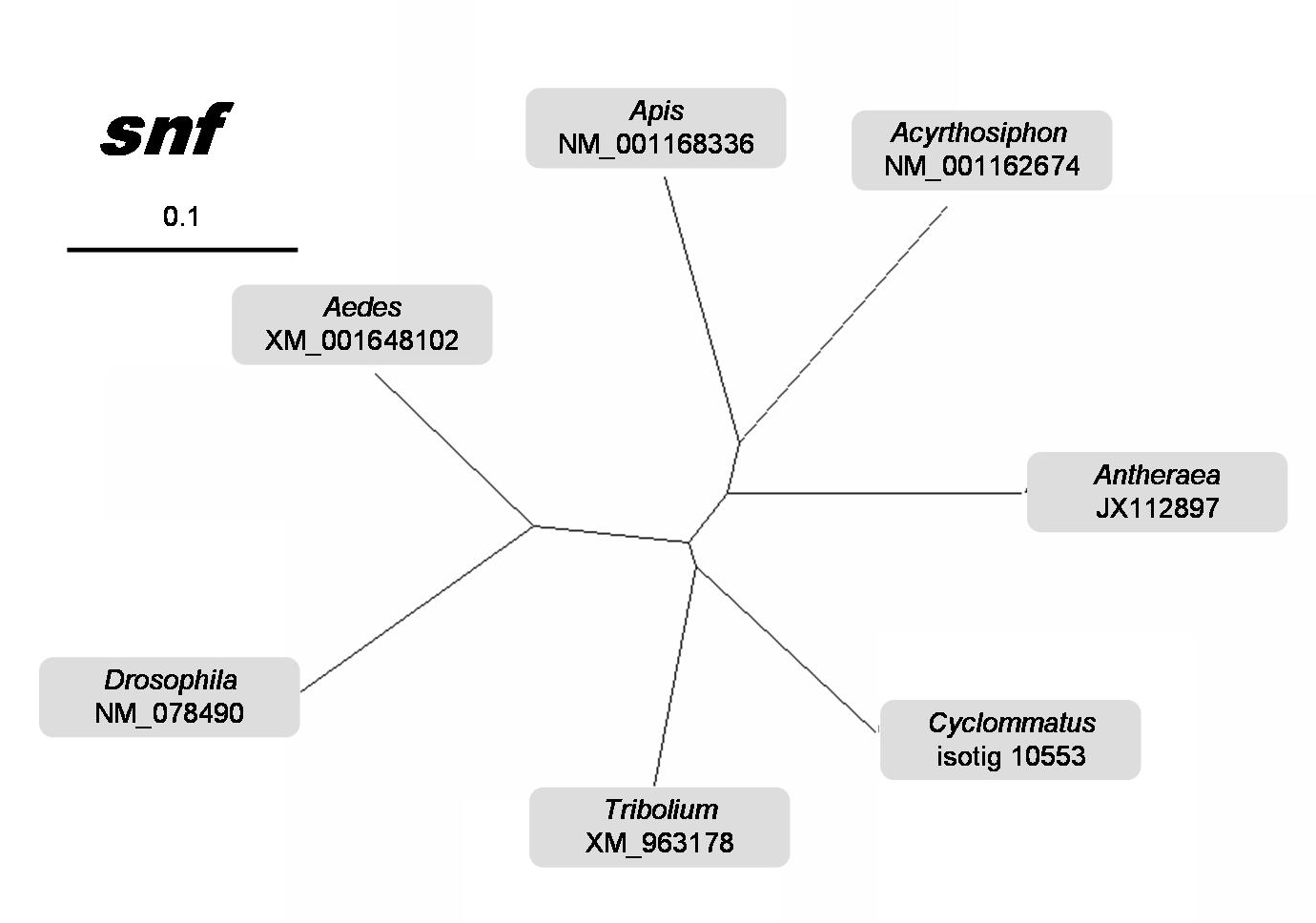


Figure S11. Unrooted phylogenetic tree of *snf*


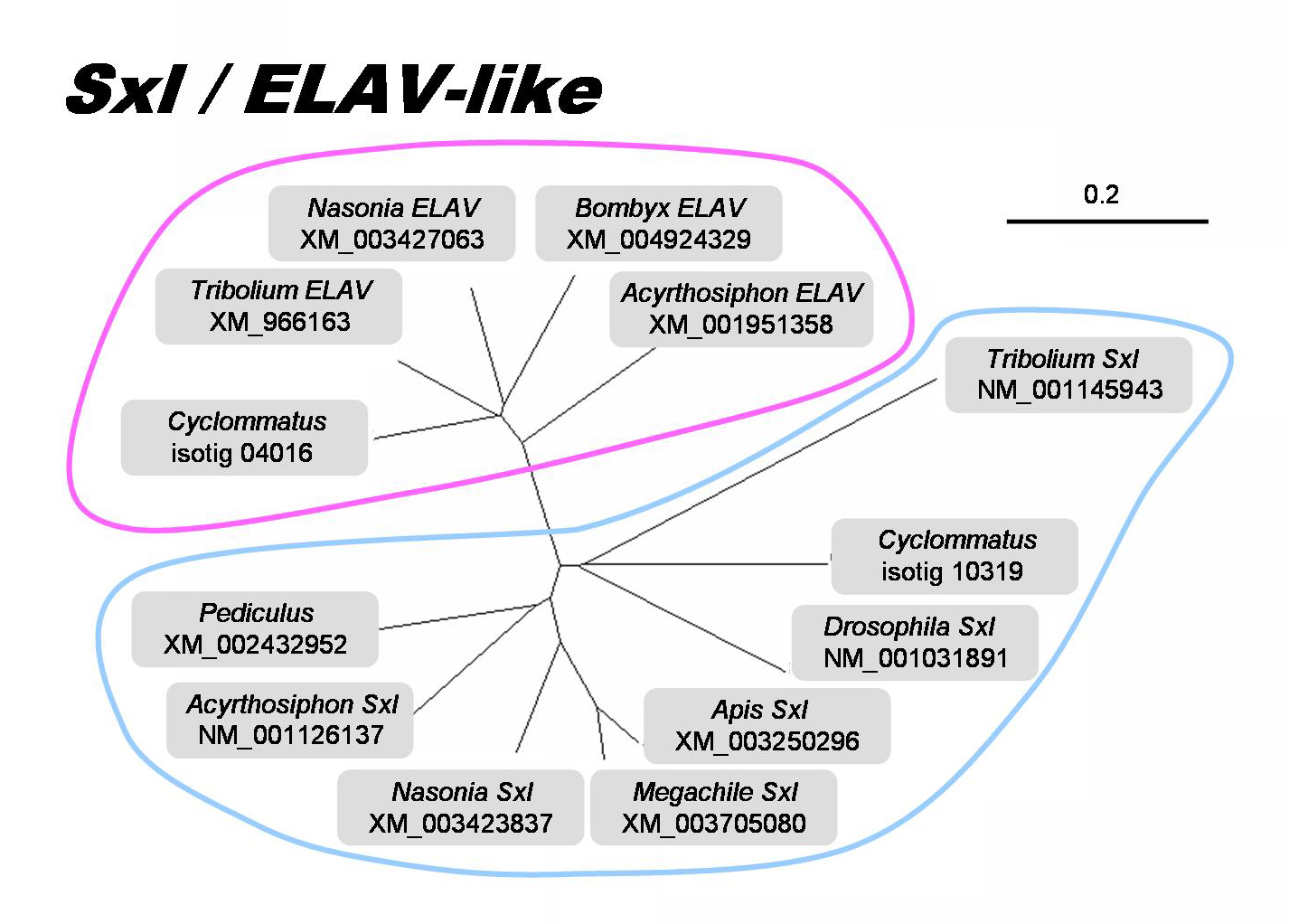


Figure S12. Unrooted phylogenetic tree of *Sxl*


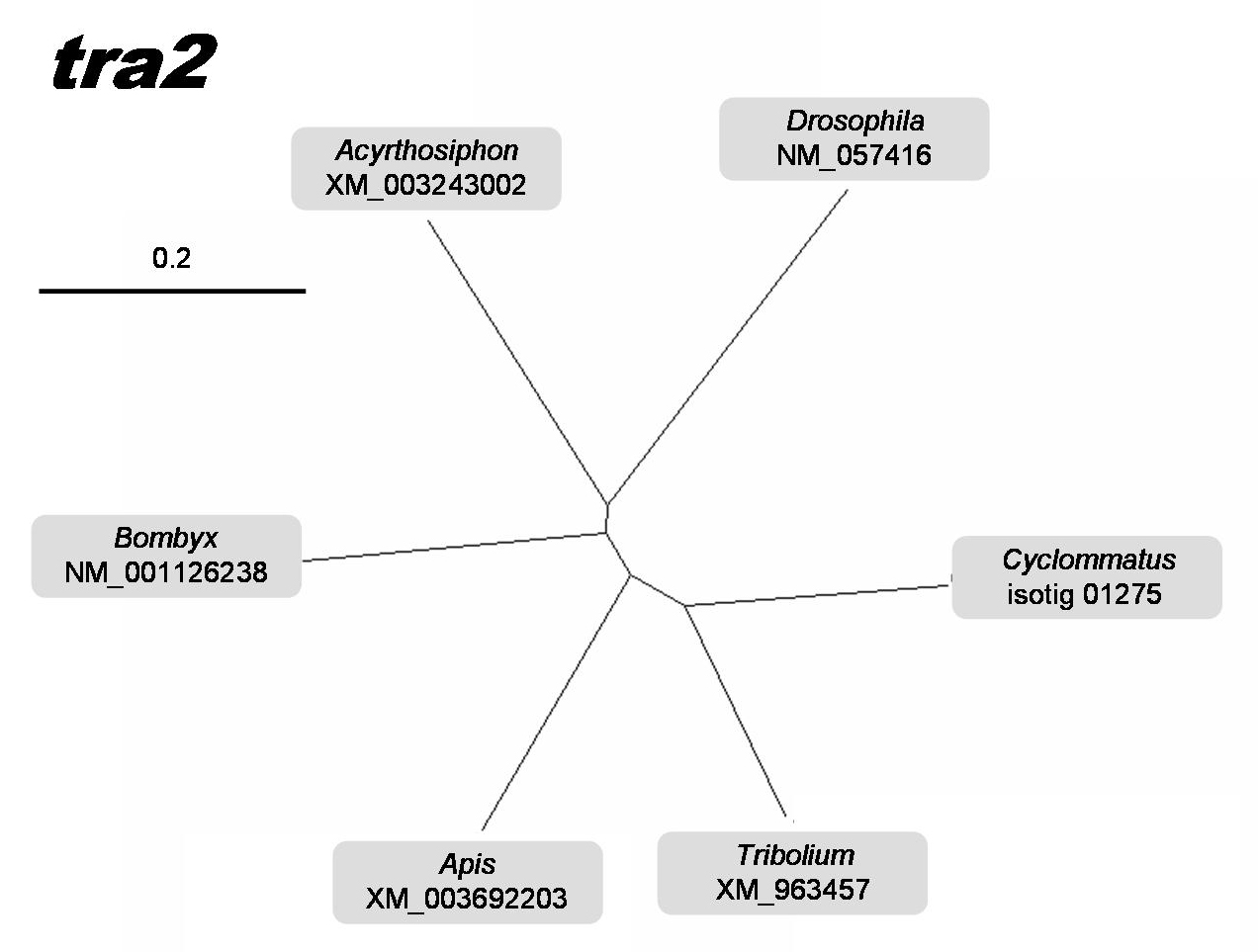


Figure S13. Unrooted phylogenetic tree of *tra2*


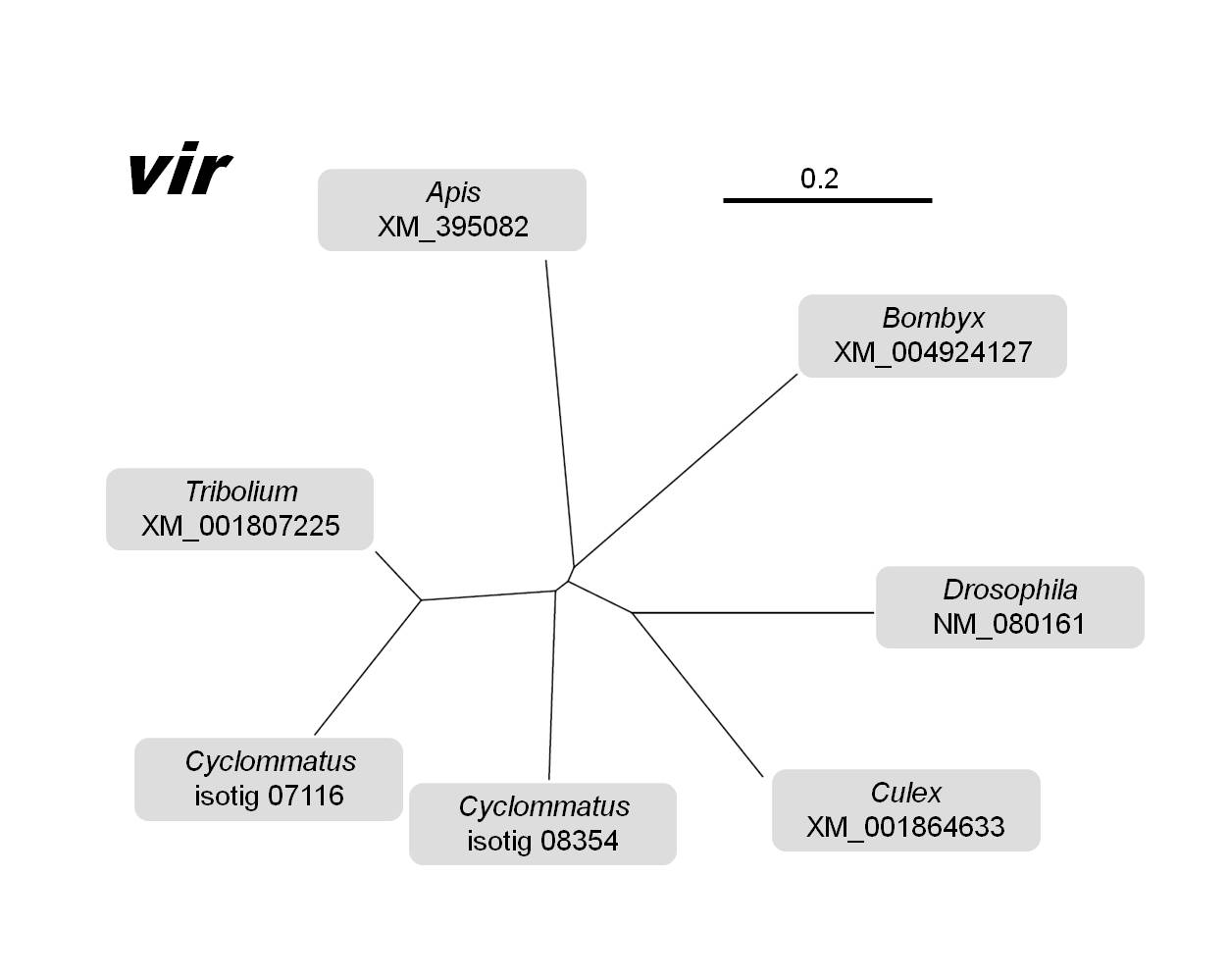


Figure S14. Unrooted phylogenetic tree of *vir*
